# Analysis of a Cas12a-based gene-drive system in budding yeast

**DOI:** 10.1099/acmi.0.000301

**Published:** 2021-12-17

**Authors:** Isabel C. Lewis, Yao Yan, Gregory C. Finnigan

**Affiliations:** ^1^​ Department of Biochemistry and Molecular Biophysics, Kansas State University, Manhattan, KS 66506, USA; ^†^​ Present address: School of Medicine, University of Texas Medical Branch, Galveston, TX 77555, USA

**Keywords:** CRISPR, Cas12a, gene drive, yeast

## Abstract

The discovery and adaptation of CRISPR/Cas systems within molecular biology has provided advances across biological research, agriculture and human health. Genomic manipulation through use of a CRISPR nuclease and programmed guide RNAs has become a common and widely accessible practice. The identification and introduction of new engineered variants and orthologues of Cas9 as well as alternative CRISPR systems such as the type V group have provided additional molecular options for editing. These include distinct PAM requirements, staggered DNA double-strand break formation, and the ability to multiplex guide RNAs from a single expression construct. Use of CRISPR/Cas has allowed for the construction and testing of a powerful genetic architecture known as a gene drive within eukaryotic model systems. Our previous work developed a drive within budding yeast using *Streptococcus pyogenes* Cas9. Here, we installed the type V *Francisella novicida* Cas12a (Cpf1) nuclease gene and its corresponding guide RNA to power a highly efficient artificial gene drive in diploid yeast. We examined the consequence of altering guide length or introduction of individual mutational substitutions to the crRNA sequence. Cas12a-dependent gene-drive function required a guide RNA of at least 18 bp and could not tolerate most changes within the 5′ end of the crRNA.

## Introduction

The discovery of a diverse set of clustered regularly interspaced short palindromic repeat (CRISPR) systems in microbes has led to the development of many molecular tools for genomic editing in basic and medical research. For the most well-studied and utilized system, the type II *Streptococcus pyogenes* Cas9 (*Sp*Cas9) has provided major advances across many types of organisms [1]. Use of this system for genomic manipulation requires the following: (i) expression of the Cas9 nuclease, (ii) a single guide RNA (gRNA) fragment and (iii) an intended DNA target that also contains a 3′ protospacer adjacent motif (PAM) with the sequence 5′-NGG-3′ (where N represents any nucleotide) [2, 3]. The power of this system lies within the programmability of the gRNA sequence – inclusion of nearly any 20 base-pair (bp) combination can be used with the universal structural RNA component (tracrRNA) and co-expressed nuclease. Together, the Cas9/gRNA complex will (i) search and bind to the corresponding DNA sequence that is complementary to the intended gRNA sequence included within the crRNA, (ii) recognize the 3′ PAM sequence within the DNA target, and (iii) create a double-strand break (DSB) at a precise position three bp upstream of the 5′ end of the PAM [4]. Eukaryotic DNA repair systems including non-homologous end joining (NHEJ) and homology directed repair (HDR) allow for subsequent editing. In the absence of any added donor DNA source, end joining causes the severed ends to be repaired, typically with an insertion or deletion occurring at the site of cleavage. On the other hand, HDR systems will allow for the inclusion of exogenous DNA (either a single base, or entire genes or constructs) provided there is sufficient flanking homology surrounding the DSB [4]. This simple, yet highly efficient methodology now allows for routine genomic alteration and editing within plants, animals, fungi and microbes.

Since the adoption of *Sp*Cas9, there has been interest in the discovery and characterization of other orthologous CRISPR systems. Many groups have pursued (i) Cas9 nucleases from different bacterial species, (ii) engineered variants developed in the lab and (iii) independent classes of CRISPR systems [5–10]. Briefly, Cas9 orthologues seem to require a unique PAM sequence and guide RNA sequence and structure [8, 11]. Modifications to existing Cas nucleases have focused on both reduction of overall protein size as well as altering PAM specificity and/or changes in potential off-target effects [6, 12]. Interestingly, the characterization of non-Cas9 nucleases has provided a new suite of molecular tools and opportunities for editing. The type V Cas12a nuclease (formally known as Cpf1) includes several major differences compared to Cas9. First, the PAM requirement is on the 5′ end of the DNA target for Cas12a and is T-rich (5′-TTN-3′) [9]. Second, the guide RNA specific for Cas12a does not include a 3′ tracrRNA structure. Rather, crRNA is flanked by short direct repeats and the nuclease is able to self-process its own guide RNA such that multiple independent (mature) guides will result from a single RNA fragment [9, 13]. Third, Cas12a creates a staggered DNA break as opposed to a blunt ended cleavage event for Cas9 [9] and this may aid in shifting a preference for DNA repair by HDR over NHEJ [14]. These and other differences provide the opportunity for potential advantages over Cas9-based editing including (i) the ability to multiplex from one gRNA expression construct, (ii) editing within AT-rich genomic regions and (iii) combinatorial applications using both type II and type V nucleases and their corresponding guide RNAs.

A unique application of CRISPR/Cas systems is within a ‘gene drive’ (GD). This biotechnology could one day be used to aid in controlling biological populations of pests, parasites and invasive species that could have profound effects on human health, agriculture and at-risk ecosystems [15–17]. The basic architecture of a CRISPR-based gene drive includes installation of the gene cassettes for the nuclease and the guide RNA at a particular genomic locus. What makes this arrangement unique is that the programmed DNA target of Cas9/gRNA will be the WT allele on the opposing homologous chromosome within a heterozygous diploid genome. In this way, a DSB will be introduced opposite of the drive locus; activation of HDR systems will use the homologous, intact chromosome to copy the entirety of the gene drive in place of the severed WT allele, thus converting the GD/WT heterozygous cell into a GD/GD homozygous cell [18–20]. This super Mendelian mechanism could theoretically allow for propagation of a drive through a global population with high speed and in a small number of generations. The intended purpose of this strategy would be to either (i) control the population through bias of sex determination causing populations to crash and be reduced or eliminated and/or (ii) to install genetic cargo proximal to the drive itself that could modify the organism (for instance, providing resistance to pathogens or altering other phenotypes). To date, these systems have been developed and studied in fungi, metazoans, bacteria and viruses under laboratory conditions [18, 19, 21–23]. Our previous work has developed a safe and ‘artificial’ drive system in budding yeast for study of various aspects of CRISPR/Cas editing such as nucleocytoplasmic trafficking, guide RNA specificity and anti-CRISPR inhibition [24–26].

In this study, we have modified our gene-drive design in *Saccharomyces cerevisiae* to include the *Francisella novicida* Cas12a (*Fn*Cas12a) nuclease gene, a corresponding guide RNA, and modified target allele system using non-native DNA sequences. We demonstrate activation of Cas12a/gRNA allows for highly efficient and successful gene-drive propagation within diploid yeast cells using a triple selection methodology. Additionally, we tested alterations to both the guide RNA length and mutational substitutions within the programmed crRNA to determine the effect on overall gene drive success. Our findings highlight that *Fn*Cas12a can edit at high levels with a variety of guide lengths, displays sensitivity to single changes within the 5′ end of the guide RNA sequence, and appears to have both positional and sequence dependent effects with regards to single nucleotide changes in the guide RNA.

## Methods

### Yeast strains and plasmids

*S. cerevisiae* strains and plasmids used in this study can be found in Tables S1 and S2 (available in the online version of this article). Haploid yeast were constructed by a combination of *in vivo* plasmid ligation [27] and subsequent chromosomal integration(s) using amplified linear PCR fragments and CRISPR/Cas12a-based editing. The design of the *Fn*Cas12a-based drive and target alleles were modelled after past iterations of *S. pyogenes* Cas9-based drives in yeast [25, 28] including a custom synthesized *Fn*Cas12a gene with a yeast codon bias (Genscript). Several alterations were also included compared to past drive systems. For instance, altered (u1) and (u2) sites [29], termed (u1′) and (u2′) were used for inclusion of the 5′ Cas12a PAM site, and a terminator sequence from the *CDC11* locus downstream of the *Schizosaccharomyces pombe HIS5* (*SpHIS5*) selection marker within the target alleles was also included (Table S1). For inclusion of the *Candida albicans URA3* (*CaURA3*) marker within the gene drive allele strains, an identical parental strain was first constructed containing the Kan^R^ cassette at the 3′ end of the *HIS3* locus. A *Fn*Cas12a guide RNA construct (pGF-V2149) was generated that targeted a sequence within the Kan^R^ gene coding strand (Fig. S1). Briefly, yeast were co-transformed with (i) the gRNA(Kan^R^) high-copy plasmid and (ii) an amplified MX-based *CaURA3* fragment followed by cell recovery within YP+GAL liquid (to activate expression of Cas12a) at 30 °C and final plating onto SD-LEU medium. Clonal isolates were assessed for (i) loss of G418 resistance (loss of the Kan^R^ allele), (ii) growth on SD-URA (inclusion of *CaURA3* allele) and (iii) sensitivity to SD-LEU (loss of guide RNA plasmid) to generate the final yeast strains. PCRs of isolated chromosomal preparations (Fig. S2) and DNA sequencing confirmed all modified loci in haploids (Fig. S1). Plasmids for this study included modifications to a parental DNA construct expressing the ‘WT’ (u1′) guide RNA sequence for *Fn*Cas12a (pGF-V1895). Site-directed mutagenesis [30] or custom gene synthesis produced the collection of expression constructs for the Cas12a guide RNAs of varying lengths and mismatches (Table S2, Fig. S1). Briefly, unique restriction sites (*SpeI*/*NotI*) were included within gRNA constructs and obtained by custom synthesis on a pUC57-based vector. The gRNA cassette was then subcloned to pRS425 (digestion of both constructs using *SpeI*/*NotI*, separation by gel electrophoresis, gel extraction and purification, *in vitro* DNA ligation, transformation into *E. coli*, plasmid extraction and DNA sequencing for confirmation). For DNA amplification via PCR, KOD Hot Start DNA Polymerase (EMB Millipore) was used with synthetic oligonucleotides (IDT). Isolation of plasmid DNA from *E. coli* was performed using a commercially available GeneJET plasmid DNA miniprep kit (Thermo Scientific); isolation of plasmid DNA from budding yeast utilized a modified protocol [27] and the same DNA extraction materials.

### Culture conditions

Yeast were grown on rich medium (YPD consisting of 2 % peptone, 1 % yeast extract and 2 % dextrose) or synthetic minimal medium (S+DEX consisting of a yeast nitrogen base, ammonium sulphate and amino acids) at 30 °C. Drop-out mixtures removed one or more amino acids (e.g. SD-URA-LEU). Raffinose and galactose were used at 2 %, while sucrose was used at 0.2 % (all sugars were filtered, not autoclaved). G418 was included in YPD plates at a concentration of 240 µg ml^−1^.

### Gene Drive assays and biosafety

Haploid strains harbouring the Cas12a drive were first transformed with the guide RNA plasmid of interest (or empty vector control) and propagated on SD-LEU-URA plates; haploids harbouring the target allele were maintained on SD-HIS medium. For gene-drive activation within diploids, haploid strains were first mated on YPD medium at 30 °C for 24 h. Next, diploids were selected by replica-plating to SD-LEU-URA-HIS plates and incubating at 30 °C for 48 h. Two additional diploid selection steps were performed on the same media type and incubated at 30 °C for 24 h. Overnight cultures of diploids were grown in S+RAFF/SUC-LEU-URA-HIS liquid medium at 30 °C with constant shaking followed by back-dilution into YP+GAL liquid for between 0–7.5 h. Cell were harvested by centrifugation, diluted into sterile water, spread onto SD-LEU-URA plates at an approximate density of 100–500 cells per plate and incubated at 30 °C for 48 h. Finally, colonies were transferred by replica-plating (sterile velvet transfer) to a new SD-LEU-URA plate and a SD-HIS plate and incubated at 30 °C for 24 h before imaging. Experiments were performed in at least triplicate. Quantification of surviving yeast colonies was done in a single-blind fashion as previously described [25, 28]. Depending on the total number of colonies per agar plate, a random sector was chosen from the SD-LEU-URA plate. The gene-drive efficiency was calculated as the percentage of colonies able to grow on SD-LEU-URA that were also sensitive to SD-HIS medium following gene-drive activation. Error was presented as the standard deviation across separate experimental trials (also see Table S4).

Our previous work has described a number of safety mechanisms built into our CRISPR/Cas9-based gene drives in budding yeast systems [24, 25]. The same principles have been adopted in this study with use of Cas12a in place of Cas9. Briefly, these include the following strategies. First, target DNA sequences and the corresponding guide RNAs are programmed to cleave non-native sequences [such as the (u1′) motif] to the *S. cerevisiae* genome to both reduce the potential for off-target effects and potential action of the drive within native populations. Second, the expression cassette for the guide RNA is present on a high-copy plasmid; this has been shown to be spontaneously lost in the absence of selection. Third, expression of the Cas12a nuclease is under control of the inducible *GAL1/10* promoter and is repressed during growth on dextrose-containing medium. Fourth, the drive locus is flanked by (u2′) sequences that can be used to self-cleave (and remove) the entire drive cassette; this can also be accomplished by targeting other non-yeast DNA within the locus (such as Kan^R^) along with donor DNA containing adequate *HIS3* flanking sequence and HDR-based repair. Fifth, there now exists a suite of anti-CRISPR proteins that evolved to directly counter and inhibit action of CRISPR/Cas nucleases such as Cas9 [24, 31, 32] and Cas12a [33, 34]. We have confirmed that several of the AcrVA proteins, when expressed in budding yeast, can inhibit *Fn*Cas12a-based gene drives (our unpublished data). Sixth, all strains, DNA and materials used with gene drive yeast were autoclaved and sterilized following experimentation.

## Results

### Design of CRISPR gene-drive system in *S. cerevisiae* using *F. novicida* Cas12a

**Fig. 1.**
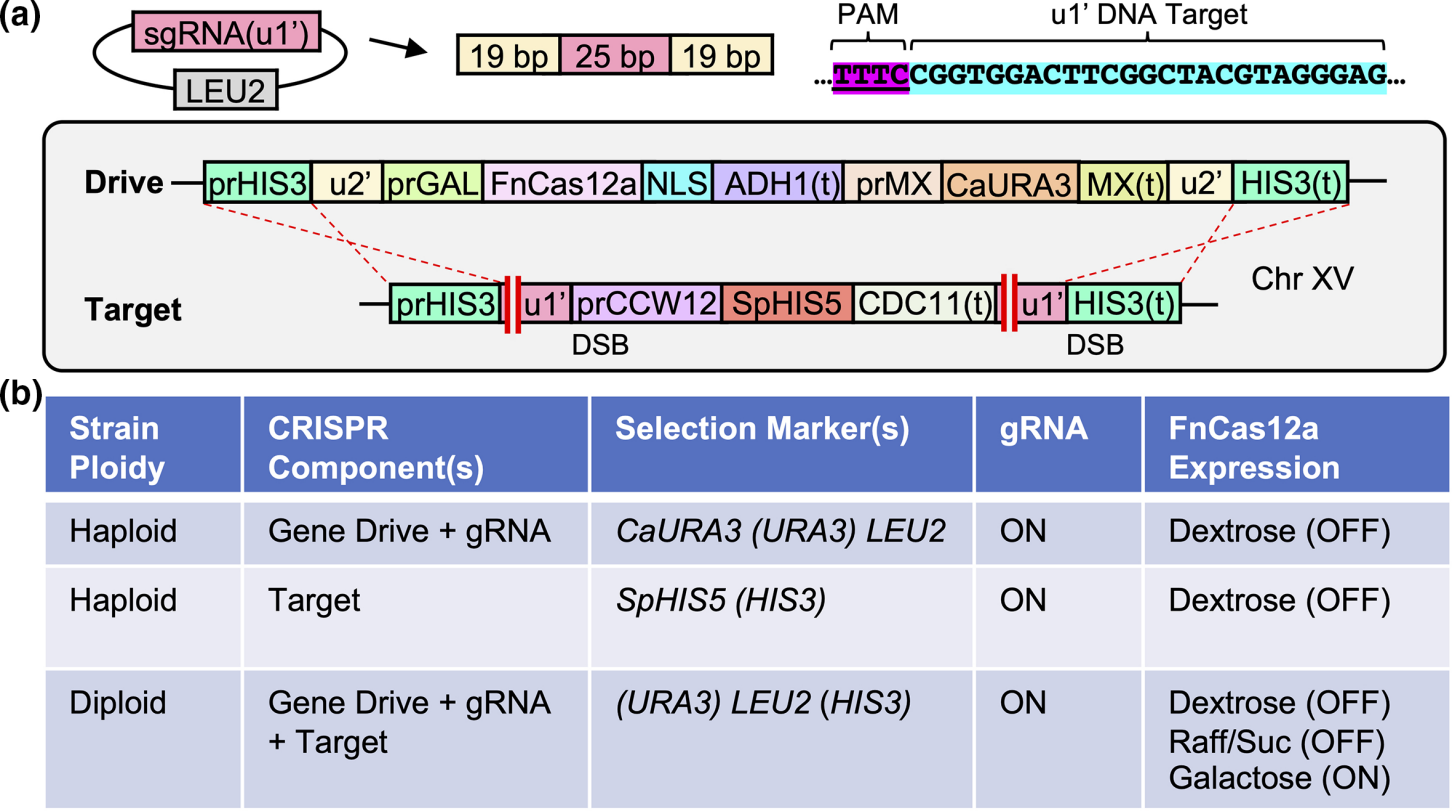
Design of a CRISPR/Cas12a-based gene-drive system in *S. cerevisiae*. (a) Artificial (u1′) and (u2′) sites were used as flanking DNA within the Cas12a drive system at the *HIS3* locus (Chromosome XV). The selection marker for the gene drive was *Candida albicans URA3*; for the target allele, *Schizosaccharomyces pombe HIS5* (functional equivalent of yeast *HIS3*) was used. An expression cassette for the *Fn*Cas12a guide RNA included 19 bp repeats flanking a crRNA of 25 bp. This high-copy plasmid included a *LEU2* selection marker. The *Fn*Cas12a gene (containing a C-terminal NLS) was under control of the inducible *GAL1/10* promoter whereas the target allele was under control of the constitutive *CCW12* promoter. (b) Table illustrating the generation and propagation of parental haploid (drive or target) strains versus final diploid (drive +target) yeast. Expression of Cas12a (unlike the guide RNA) was restricted to the diploid strain (galactose). Also, selection was constantly maintained for both the guide RNA plasmid (*LEU2*) and the drive allele (*URA3*) until activation of the nuclease in rich medium with galactose.

Our previous work in yeast has employed use of the type II *S. pyogenes* Cas9 to power gene-drive systems within diploid cells [24–26, 28]. Given the discovery and widespread availability of other CRISPR nucleases and engineered variants, we predicted that use of alternative systems may also allow for successful gene-drive (GD) activity *in vivo*. Therefore, we altered our previous GD constructs to include (i) the type V *F. novicida* Cas12a (formally Cpf1) nuclease, (ii) a corresponding guide RNA (gRNA) and (iii) appropriate target DNA sequences (Fig. 1). The *Fn*Cas12a gene (translated to 1300 residues) was codon optimized for expression in yeast including a C-terminal nuclear localization signal (NLS). The guide RNA for Cas12a included several important differences compared to the guide RNA for Cas9. First, there is no extended tracrRNA sequence at the 3′ end of the crRNA sequence; rather, a short repeat is found at the 5′ end of the crRNA. Previous work with Cas12a including studies in yeast [35] determined that inclusion of a flanking direct repeat of 19 bp placed on either side of the crRNA is sufficient for successful editing *in vivo*. Moreover, Cas12a is involved in processing of pre-crRNA fragments resulting in a 5′ direct repeat upstream of the crRNA for subsequent editing of target DNA. Finally, rather than requiring a PAM sequence of 5′-NGG-3′ at the 3′ end of the DNA target (such as for *Sp*Cas9), the *Fn*Cas12a nuclease required a PAM sequence 5′ of the target DNA with the sequence 5′-TTTV-3′ (V is any nucleotide other than thymine) [35].

To accommodate these differences, we modified the existing architecture of our previous gene-drive system in yeast to include the sequence 5′-TTTC-3′ directly upstream of the unique (u2) or (u1) sites flanking the nuclease expression cassette or target allele, respectively, to create (u2′) and (u1′) (Figs 1 and S1). We have previously demonstrated the utility and importance of targeting artificial DNA sequences when studying gene drives *in vivo* [25]. Briefly, use of a programmed non-native sequence [29] allows for (i) multiplexing with one guide RNA, (ii) complete excision of target genes (including selection markers), (iii) potential reduction of off-target effects, (iv) rapid self-excision of the nuclease gene if needed as a safeguard, and (v) prevention of inappropriate activation of the gene drive in case of theoretical release since the target sequence(s) do not exist in native *S. cerevisiae* genomes. We chose to synthesize a guide RNA expression cassette (RNA Pol III) developed by previous groups [36] that included 19 bp direct repeats flanking a crRNA of 25 bp. We also modified the target allele(s) at the *HIS3* locus to include a much shorter gene cassette with a unique terminator sequence (compared to previous iterations). Finally, our gene-drive design standardized all selection markers within the drive (*URA3*), guide RNA (*LEU2*) and target (*HIS3*) to be compatible auxotrophic markers. Prior to expression of Cas12a using galactose medium, our system maintained active repression of the nuclease, constitutive expression of the guide RNA, and maintenance of both the drive and target alleles at the *HIS3* locus through the diploid selection process (Fig. 1b).

### An efficient and successful Cas12a-based gene drive system in yeast

**Fig. 2.**
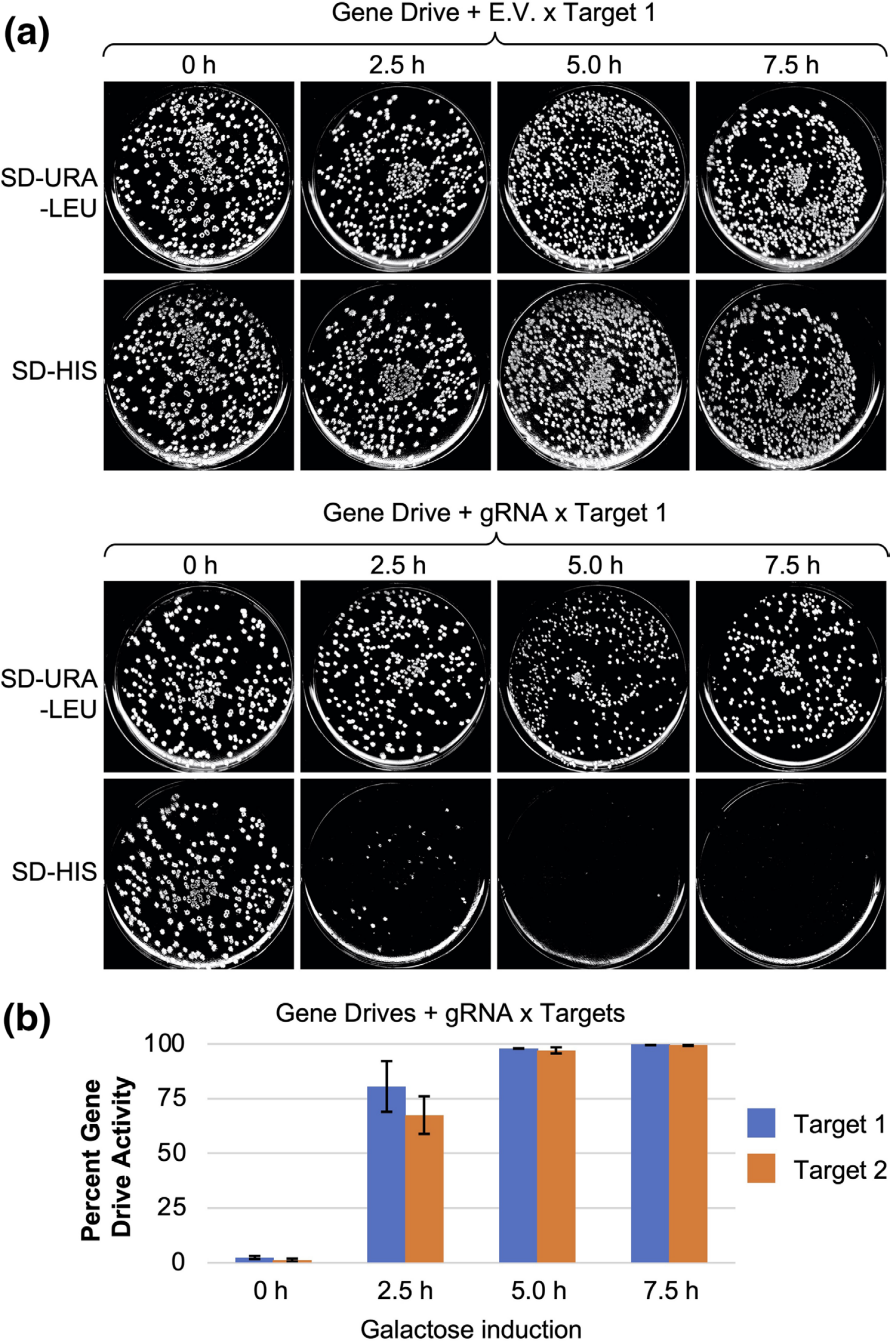
An efficient Cas12a-based gene drive *in vivo*. (a) The haploid strain GFY-4625 (gene drive) was transformed with a plasmid containing the guide RNA plasmid (pGF-V1895) to target the (u1′) DNA sites or an empty vector (pRS425). The gene drive assay included (i) mating the drive strains with haploids of GFY-4424 (target), (ii) selecting diploids, (iii) culturing diploids in S+RAFF/SUC-LEU-URA-HIS liquid overnight, (iv) diluting cells in YP+GAL for between 0–7.5 h and (v) plating yeast onto SD-LEU-URA plates. Finally, viable colonies were transferred to SD-LEU-URA and SD-HIS plates by replica-plating and imaged after 24 h of growth (also see Fig. S3 for unmodified images). (b) Gene-drive activation was performed for three independent drive strains (GFY-4625, GFY-4626 and GFY-4627) and two separate haploids containing targets (target 1, GFY-4424; target 2, GFY-4425) for a similar time course as performed in (a). Surviving colonies were quantified to illustrate the percent gene-drive activity (the fraction of colonies viable on SD-LEU-URA but sensitive to the SD-HIS condition). The average of three separate drive strains is shown (*n*>100 colonies per sample). Error, sd.

Our gene-drive system included placement of the nuclease (expression inhibited) and the guide RNA-containing plasmid within one haploid yeast strain and the intended target DNA at the same locus within a second haploid yeast strain of the opposite mating type. Activation of the system involved mating the strains together and selecting for only diploid cells (also harbouring the guide RNA plasmid). Finally, cultures of diploids were incubated in galactose for varying amounts of time to activate expression of Cas12a (along with constitutive expression of the guide RNA). Cells were then plated onto SD-URA-LEU medium and incubated, allowing for growth of individual visible colonies. Finally, yeast were transferred onto both a SD-URA-LEU and a separate SD-HIS plate. Diploid cells that (i) expressed Cas12a, (ii) targeted the (u1′) site(s) flanking the target allele, and (iii) copied the drive locus in place of the target locus would be sensitive to the SD-HIS condition. This experiment was performed for drive-containing haploids with an empty plasmid or the guide RNA to the target sites (Figs 2a and S3). Yeast with the empty plasmid displayed robust growth on SD-HIS medium following galactose culturing. However, inclusion of the guide RNA plasmid caused a distinct and similar pattern of growth as seen with Cas9-containing yeast gene drives. Increasing the time cultured in galactose resulted in a marked decrease in surviving colonies on SD-HIS (Fig. 2a). The experiment was repeated in triplicate with two variations of the target allele (Fig. S1); we observed that 5 h in galactose resulted in nearly 100 % of the colonies sensitive to the SD-HIS condition, suggesting a loss of the *SpHIS5* target allele and replacement by the drive allele through HDR.

**Fig. 3.**
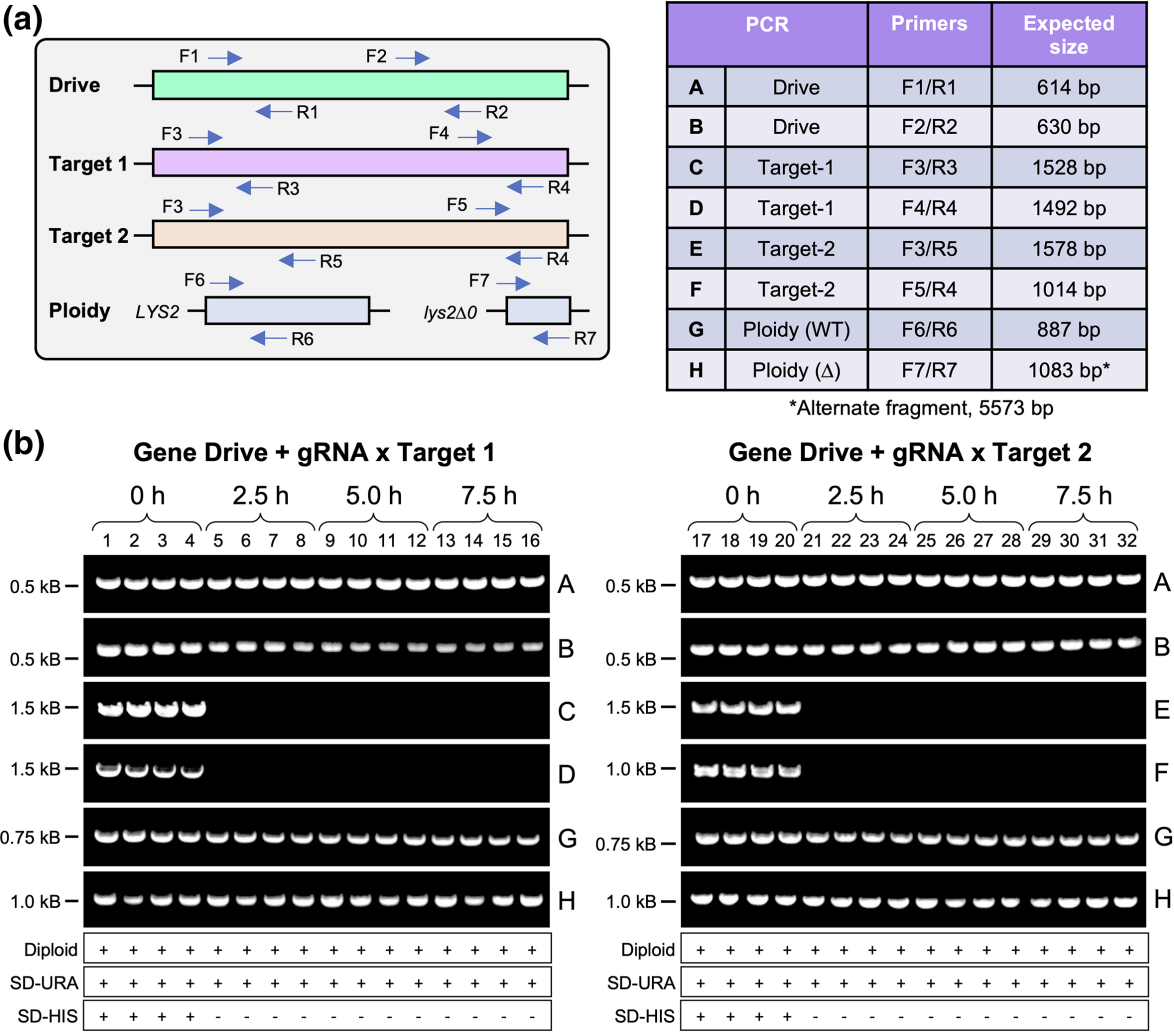
Characterization of gene-drive success within diploids using diagnostic PCR. Following gene-drive activation of diploids (Fig. 2), four random clonal isolates were obtained from each time point for the gene-drive strain crossed to either haploid target 1 or target 2 for further analysis. (a) Left: chromosomal DNA was isolated and purified for each diploid strain and tested by PCR across both the *HIS3* and *LYS2* loci. Oligonucleotides used in this study can be found in Table S3. Right: the expected product size(s) for each diagnostic PCR. (b) Strains were also retested on SD-URA and SD-HIS plates for survival. Diploid status was determined through amplification of *LYS2* (PCRs G, H). PCRs were separated by DNA gel electrophoresis and visualized. Distinct DNA gels are separated by white lines; the nearest molecular marker is illustrated on the left. PCRs C and D were only performed for target 1 (left) whereas PCRs E and F were only performed for target 2 (right). Unmodified images can be found in Fig. S4.

To verify that the gene-drive plating assay was accurately illustrating Cas12a-based editing of the target allele followed by replacement of the target locus using the drive allele, we utilized a collection of diagnostic PCRs to assay both the *HIS3* locus (drive and/or target) as well as the *LYS2* locus. The parental haploid strains used in this study (BY4741, *MAT* **a** and BY4742**,** *MAT⍺*) differ at both the *MET15* and *LYS2* loci (Table S1). PCR amplification of the *LYS2* locus served as a convenient method to determine if isolated clones from the gene drive assay (presumably diploids from the selection protocols) contained both a WT and a deleted copy of *LYS2* (haploids would only harbour one or the other allele, not both). For both the *HIS3* and *LYS2* loci, two independent PCRs were performed using unique oligonucleotide pairs (Fig. 3a, Table S3). Following gene-drive activation, clonal isolates were randomly selected from the SD-URA-LEU plate for all conditions (Fig. 2b) and retested for growth on either SD-URA (drive selection) or SD-HIS (target selection) (Fig. 3b). We performed PCRs on isolated and purified chromosomal DNA of four random clones from each time point for experiments using the target 1 strain (Fig. 3b, left) or target 2 strain (Fig. 3b, right). Prior to induction in galactose, DNA from isolates allowed for the amplification of all six PCR products, suggesting the presence of both the drive and target alleles as well as both versions of the *LYS2* locus (Fig. 3b). Following activation of Cas12a, all isolates tested were unable to successfully amplify fragments of the expected size at the target *HIS3* locus and this correlated with sensitivity to the SD-HIS condition (Figs 3b and S4). Moreover, all tested yeast appeared to be diploids and allowed for amplification of both alleles of *LYS2*. Together, these data suggest that Cas12a and its guide RNA allow for a highly efficient gene-drive system in budding yeast diploid cells.

### Robustness of Cas12a-based gene drives to alterations in guide RNA sequence

**Fig. 4.**
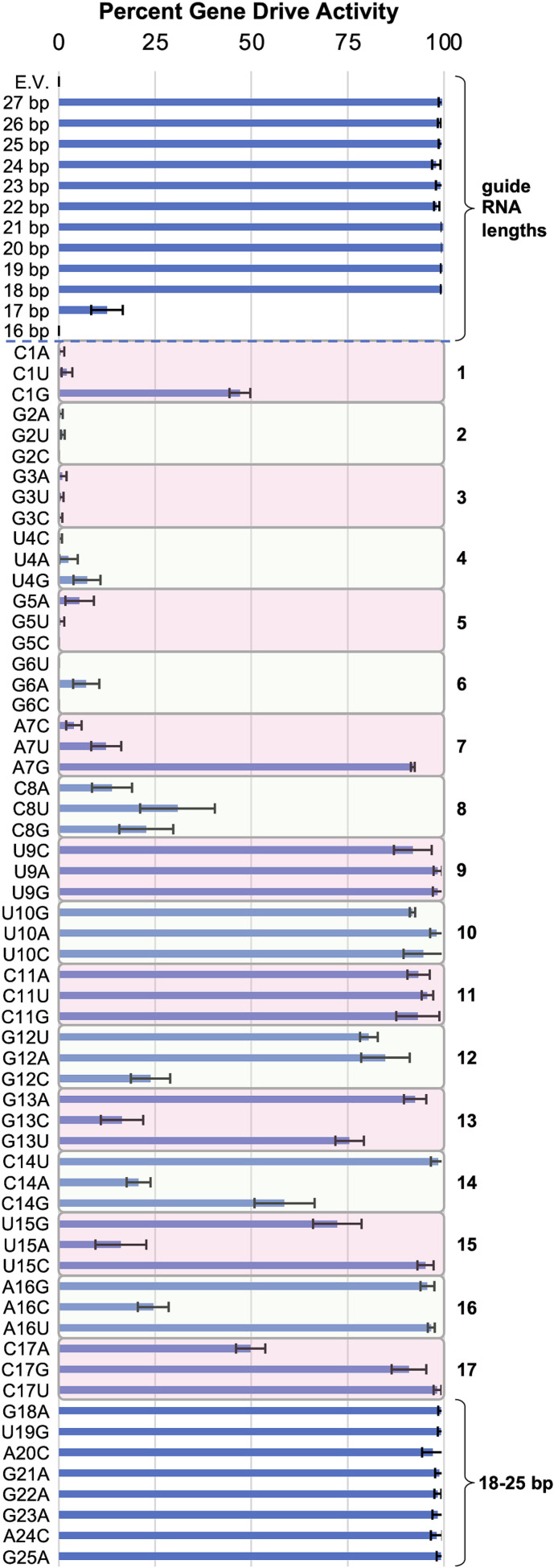
Tolerance of the Cas12a gene-drive system to alterations in guide RNA length and RNA/DNA mismatches. Three gene-drive strains (GFY-4625, GFY-4626, GFY-4627) were transformed with an identical set of guide RNA or control plasmids (72 total; see Table S2) and crossed with haploid GFY-4424 to generate diploids. Following Cas12a activation, strains were quantified for the percent gene-drive activity as in Fig. 2(b) (also see Table S4). The average of the three strains is presented; error is standard deviation. Top: the guide RNA plasmid was altered to present a final crRNA size of 16–27 bp (flanked by the 19 bp repeat). Note, the guide RNA length of 27 bp only contains a full RNA/DNA match (27/27) to the downstream (u1′) target site as the upstream site contains a 3′ mismatch (26/27). Bottom: a guide RNA of 25 bp targeting the (u1′) sequence was used. Nucleotide position is numbered 1–25 beginning at the 5′ end of the crRNA sequence. For the 17 nucleotides at the 5′ end, each base was mutated to one of the three possible alternatives (RNA change is displayed). For positions 18–25, only a single bp change was tested. For most conditions, >100 colonies were quantified.

Numerous studies have demonstrated that editing efficiency with CRISPR/Cas is dependent on guide RNA sequence, length, mismatches, structure and even chemical modifications [9, 37–39]. We chose to examine how alteration of the crRNA sequence length used to target the flanking (u1′) sites within our system would affect overall gene-drive efficiency. We tested guide RNAs of 16–27 bp (extending or reducing the sequence at the 3′ end) and found nearly 100 % drive activity for guide RNAs of lengths 18–27 (Fig. 4, top). There was a low level of activity for a guide RNA length of 17 bp, whereas no activity was seen for a guide length of 16 bp. This appears similar to other findings [9] where a range of guide lengths could provide efficient editing. Of note, all guide RNA expression cassettes maintained identical flanking 19 bp direct repeats (Fig. S1).

Next, we examined the consequence of a single base mismatch within the crRNA sequence of a guide RNA of 25 bp in total length. For completeness, we mutated each base within the guide RNA expression cassette to all possible nucleotides for positions 1–17 (counting from the 5′ end of the crRNA). For positions 18–25, only a single substitution was tested. These experiments were done in triplicate, using three independent drive strains and performed at the same time as the guide length experiments. Including a negative control strain harbouring an empty vector, these 72 separate gene-drive haploids were mated to the target strain, selected for diploids, activated by culturing in galactose, plated and quantified for activity (Fig. 4, bottom, Table S4). We observed several trends that defined regions within the crRNA sequence. First, substitutions within positions 1–8 resulted in a near total loss of activity apart from the C1G and A7G mutants (high activity) and all C8 substitutions (low activity). Second, there appeared to be little effect from any substitution within positions 9–11. Third, for positions 12–17, an interesting pattern seemed to repeat – two substitutions provided relatively high editing whereas one substitution caused a marked decrease or low editing. It may include sequence-specific changes as both G12C and G13C caused reduced editing as well as both C14A and C17A; it remains unclear if the same trend exists for uracil or adenine mutations such as U15A and A16C, both of which had the strongest reduction on overall editing and drive activity. Finally, no change was seen for mutations within positions 18–25. These data illustrate that (i) critical nucleotides within the crRNA include the first eight at the 5′ end and this appears independent of the identity of the substitution, (ii) a central region of the crRNA (defined by positions 9–11) may not be critical for editing within the context of a 25 bp guide, (iii) positions 12–17 may represent a region where Cas12a/gRNA function is dependent on the nature of the substitution, and (iv) the 3′ region consisting of positions 18–25 have little individual contribution to overall editing success and this is supported by our observations of guide RNAs of varying lengths.

## Discussion

### Alternative CRISPR nucleases to power gene drives

Current gene-drive biotechnology utilizes a nuclease (most commonly, *S. pyogenes* Cas9) to create a double-strand break at a particular location within the genome. Subsequent DNA repair via HDR allows the non-targeted homologous chromosome to serve as the donor DNA and copies the entirety of the drive locus itself (and any associated cargo DNA), thus converting a heterozygous condition into a homozygous condition for the allele(s) of interest. Since the primary purpose of the (active) nuclease, when paired with appropriate guide RNA(s), is to introduce a DSB, it was expected that a drive system could include the type V *Fn*Cas12a nuclease and allow for proper drive conversion *in vivo*. Use of alternative nucleases and engineered variants of CRISPR nucleases (including Cas12a from other species) will allow for the development of more complex drive systems in the future. For instance, due to the difference in PAM specificity between Cas9 (5′-NGG-3′) and Cas12a (5′-TTN-3′), the choice of which nuclease to include within a gene drive may depend on the GC-content of either the locus of interest, or the prevalence across a genome. Moreover, we envision that complex drives could co-exist within the same genome – our previous work has demonstrated that a triple-locus drive is possible with three separate guide RNAs [40]. However, in that arrangement, a single copy of *Sp*Cas9 was expressed and used to independently target each locus with a unique guide RNA [39]. A modified system might include two or more nucleases, each with a unique guide RNA construct, to selectively target one or more loci within a genome. This would provide additional control over selectivity of drive function and modulation or inhibition (if needed) using, for instance, competing ‘anti-drives’ [41] or introduction or induction of anti-CRISPRs [24]. The ability to selectively activate (or repress) multiple independently functioning nucleases might also be applied to use of enzymatically ‘dead’ variants (such as dCas9) [42] in order to modify transcription of distal gene targets unrelated to the primary function of DSB introduction to propagate the drive itself. Finally, use of two or more nucleases might provide additional redundancy to ensure DSB formation at a particular locus with independent targets within a single gene of interest. Along these lines, recent work has successfully engineered a ‘fusion guide RNA’ construct that can be used by both Cas9 and Cas12a [43].

### Guide RNA mismatch specificity

Our analysis of substitutions to the guide RNA for *Fn*Cas12a illustrate the importance of nucleotides at the 5′ end; other studies of Cas12a have also suggested the first few bases of the ‘seed’ region (PAM proximal bases required for full editing) are critical for function [9, 13, 44–47]. While numerous studies have investigated the effects of mutational substitutions (or additions or deletions) within the crRNA guide sequence for a variety of CRISPR nucleases, it is important to highlight that our assay is distinct from previous efforts for a number of reasons: (i) there are dual identical (u1′) sites at the target locus and (ii) the measurement of ‘gene drive activity’ corresponds to loss of the selection marker and is not a direct measure of DNA cleavage. Our previous work has demonstrated that in a yeast drive system, NHEJ may occur at an extremely low level (if at all) [28]. Therefore, we attribute the ‘success’ of the drive (via loss of the target locus) to the combination of Cas12a/gRNA binding, recruitment to the DNA target(s), DSB formation, and finally, repair via HDR within a diploid genome. Therefore, our findings for guide RNA requirements may differ from either *in vitro* or *in vivo* studies (typically done through plasmid DNA linearization or NHEJ-based repair and detection, respectively). For technical reasons, we chose to introduce the mutational substitution within the guide RNA expression cassette and not within the yeast genome. Our approach created a gRNA(mutated)/DNA(WT) pairing. Future work will be required to determine if similar results would be obtained through the same substitutions within the genome to create a gRNA(WT)/DNA(mutated) combination.

We cannot rule out the possibility that guide RNA sequence alterations reduced RNA stability, mature crRNA processing, binding to Cas12a and/or RNA/DNA hybridization at the target sequence. Previous work has suggested that sequences within the programmable crRNA may disrupt secondary structure formation of the direct repeat and subsequent processing of the guide RNA or binding to Cas12a [48]. Moreover, a similar observation was reported for Cas9 where interactions between bases within the crRNA could impede nuclease function [49].

Our findings from this study suggest that mismatches within the *Fn*Cas12a guide RNA to the target genome may be both positional and sequence dependent. Our experiments did not investigate whether additional alterations to the guide RNA (consecutive mutations, deletions, additions, etc.) would exacerbate the effects of a single substitution, as has been performed by other groups [44, 46, 50]; commonly, the presence of multiple mutations greatly disrupts editing. Along these lines, our analysis was limited to only the programmed artificial [u1′] target sites; it remains to be seen whether similar results will be found at different genomic loci and/or using distinct DNA sequences as targets. Extensive studies on *S. pyogenes* Cas9 guide RNA substitutions demonstrated variability across independent loci [37]; however, it may be difficult to separate the effects of genomic position versus sequence without use of an identical target sequence that either naturally exists across loci or has been engineered to include identical targets (as has been done in our yeast system). While our study has provided a preliminary investigation of guide RNA requirements for *Fn*Cas12a, future work will be required to validate whether positions within the guide, specific nucleotide substitutions and/or potential RNA secondary structure(s) can be predicted to reduce or eliminate editing given the chosen sequence and genomic context.

### Gene drives and evolved resistance

It has been well documented that there are multiple sources that can directly inhibit successful action of a gene drive [51–55]. Briefly, this corresponds to alteration of the target DNA sequence through various mechanisms: (i) natural variation within a population that includes mismatches to the guide RNA, (ii) *de novo* mutation at the target DNA site, and/or (iii) destruction or elimination of the target site through NHEJ-based repair following successful DSB formation. Therefore, different strategies are being employed to potentially reduce and/or eliminate drive resistance. These include use of multiplexed guide RNAs targeting separate DNA positions, choice of the gene target, choice of conserved DNA sites, and novel drive architectures [56–58]. Understanding how DNA polymorphisms might affect CRISPR/Cas-based DSB formation at intended target site(s) may provide additional resources to combat potential drive resistance. This could include installation of a small gRNA library harbouring potential substitutions for a high-priority DNA target to buffer against possible mutation or unknown genetic variability at the site. A more complete understanding of Cas/gRNA recognition, sequence preferences, and mismatch tolerance would assist in the design and study of safe, controllable and effective gene-drive systems.

### Data availability statement

Yeast strains and/or DNA plasmids used in this study will be made available for research or educational purposes upon reasonable request.

## Supplementary Data

Supplementary material 1
