# Supplementary material for: Analysis of a Cas12a-based gene-drive system in budding yeast
Source: Access Microbiol. 2021 Dec 17;3(12):000301. doi: 10.1099/acmi.0.000301 (PMC8749140; doi:10.1099/acmi.0.000301)
Supplement: Supplementary material 1 [file acmi-3-0301-s001.pdf]

SUPPLEMENTAL TABLES and FIGURES for

Analysis of a Cas12a-based gene drive system in budding yeast

Isabel Lewis[1,2], Yao Yan[1], and Gregory C. Finnigan[1†]

[1]Department of Biochemistry and Molecular Biophysics,

Kansas State University, Manhattan, KS 66506 USA

Running Title: Cas12a gene drive in yeast

[2]Current Address: School of Medicine, University of Texas Medical Branch, Galveston, TX, 77555 USA

[†]Correspondence to:

Gregory C. Finnigan

Dept. of Biochemistry & Molecular Biophysics

Kansas State University

141 Chalmers Hall, 1711 Claflin Rd.

Manhattan, KS 66506 USA

Phone: (785) 532-6939; FAX; (785) 532-7278;

E-mail: gfinnigan@ksu.edu

Table S1. Yeast strains used in this study.

**Strain**	**Genotype**	**Reference**
BY4741	*MAT***a** *his3Δ1 leu2Δ0 met15Δ0 ura3Δ0 LYS2*	[1]
BY4742	*MATα his3Δ1 leu2Δ0 lys2Δ0 ura3Δ0 MET15*	[1]
GFY-4424[a]	BY4742;<br>*his3Δ::prHIS3::(u1')::prCCW12::SpHIS5::CDC11(t)::(u1')::HIS3(t)*	This study
GFY-4425[a]	BY4742;<br>*his3Δ::prHIS3::(u1')::prCDC12::mCherry::SHS1(t)::prCCW12::*<br>*SpHIS5::CDC11(t)::(u1')::HIS3(t)*	This study
GFY-4625[b],<br>GFY-4626,<br>GFY-4627	BY4741;<br>*his3Δ::prHIS3:(u2')::prGAL1/10::FnCas12a::NLS::ADH1(t):prMX::Ca*<br>*URA3::MX(t)::(u2')::HIS3(t)*	This study

[a]The (u1') sites included the sequence 5'-**TTTC**CGGTGGACTTCGGCTACGTAGGGAGT-3'. The *Fn*Cas12a PAM sequence (5'-TTTV-3') is bold and underlined. Note, in strain GFY-4424, there was a "C" downstream of the target site (the 27th base) that was placed upstream of pr*CCW12* whereas there was a "G" downstream of the target site (27th base) following *CDC11*(t). For strain GFY-4425, the 27th base was a "G" within both (u1') sites. These two strains were modeled after GFY-3733 [2], but replaced the *MX*(t) with the *CDC11*(t) sequence; GFY-4424 was also considerably reduced in size by removing the cargo gene cassette.

[b]GFY-4625, 4626, and 4627 are three separately constructed and confirmed gene drive haploid strains. The included (u2') sites were 5'-**TTTC**GCTGTTCGTGTGCGCGTCCTGGGAGT-3'. Briefly, a parental strain (GFY-3798) was first constructed that harbored the pr*MX-Kan*^{R}*-MX*(t) marker cassette downstream of *ADH1*(t). Next, yeast were cultured in synthetic complete medium with raffinose and sucrose and the guide RNA plasmid (pGF-V2149) was co-transformed with a PCR fragment of the pr*MX-CaURA3-MX*(t) cassette (from pJT2869). Third, yeast were recovered overnight in YP+GAL and plated onto SD-LEU. Clonal isolates were selected prior to testing on SD-URA. Subsequent growth on YPD plates allowed for spontaneous loss of the *LEU2*-based sgRNA vector. Final strains were confirmed by sensitivity to G418, sensitivity to SD-LEU, and growth on SD-URA. PCRs of chromosomal DNA preparations followed by DNA sequencing confirmed the entire modified *HIS3* locus.

Table S2. Plasmids used in this study.

Plasmid	Description	Guide Length (bp)	Reference
pRS425	2μ, *LEU2*	N/A	[3]
pGF-V1941	pRS425; *prSNR52::Fn-sgRNA(u1'-WT)::SUP4(t)*	27	This study
pGF-V1942	pRS425; *prSNR52::Fn-sgRNA(u1'-WT)::SUP4(t)*	26	This study
pGF-V1895[a]	pRS425; *prSNR52::Fn-sgRNA(u1'-WT)::SUP4(t)*	25	This study
pGF-V1943[b]	pRS425; *prSNR52::Fn-sgRNA(u1'-WT)::SUP4(t)*	24	This study
pGF-V1944	pRS425; *prSNR52::Fn-sgRNA(u1'-WT)::SUP4(t)*	23	This study
pGF-V1945	pRS425; *prSNR52::Fn-sgRNA(u1'-WT)::SUP4(t)*	22	This study
pGF-V1946	pRS425; *prSNR52::Fn-sgRNA(u1'-WT)::SUP4(t)*	21	This study
pGF-V1947	pRS425; *prSNR52::Fn-sgRNA(u1'-WT)::SUP4(t)*	20	This study
pGF-V1948	pRS425; *prSNR52::Fn-sgRNA(u1'-WT)::SUP4(t)*	19	This study
pGF-V1949	pRS425; *prSNR52::Fn-sgRNA(u1'-WT)::SUP4(t)*	18	This study
pGF-V1950	pRS425; *prSNR52::Fn-sgRNA(u1'-WT)::SUP4(t)*	17	This study
pGF-V1951	pRS425; *prSNR52::Fn-sgRNA(u1'-WT)::SUP4(t)*	16	This study
pGF-V1952[c]	pGF-V1895; C1A	25	This study
pGF-V2210	pGF-V1895; C1T	25	This study
pGF-V2211	pGF-V1895; C1G	25	This study
pGF-V2053	pGF-V1895; G2A	25	This study
pGF-V2212	pGF-V1895; G2T	25	This study
pGF-V2213	pGF-V1895; G2C	25	This study

pGF-V1953	pGF-V1895; G3A	25	This study
pGF-V2214	pGF-V1895; G3T	25	This study
pGF-V2215	pGF-V1895; G3C	25	This study
pGF-V2054	pGF-V1895; T4C	25	This study
pGF-V2216	pGF-V1895; T4A	25	This study
pGF-V2217	pGF-V1895; T4G	25	This study
pGF-V1954	pGF-V1895; G5A	25	This study
pGF-V2218	pGF-V1895; G5T	25	This study
pGF-V2219	pGF-V1895; G5C	25	This study
pGF-V2055	pGF-V1895; G6T	25	This study
pGF-V2220	pGF-V1895; G6A	25	This study
pGF-V2221	pGF-V1895; G6C	25	This study
pGF-V1955	pGF-V1895; A7C	25	This study
pGF-V2222	pGF-V1895; A7T	25	This study
pGF-V2223	pGF-V1895; A7G	25	This study
pGF-V2056	pGF-V1895; C8A	25	This study
pGF-V2224	pGF-V1895; C8T	25	This study
pGF-V2225	pGF-V1895; C8G	25	This study
pGF-V1956	pGF-V1895; T9C	25	This study
pGF-V2065	pGF-V1895; T9A	25	This study
pGF-V2066	pGF-V1895; T9G	25	This study
pGF-V2057	pGF-V1895; T10G	25	This study

pGF-V2071	pGF-V1895; T10A	25	This study
pGF-V2072	pGF-V1895; T10C	25	This study
pGF-V1957	pGF-V1895; C11A	25	This study
pGF-V2067	pGF-V1895; C11T	25	This study
pGF-V2068	pGF-V1895; C11G	25	This study
pGF-V2058	pGF-V1895; G12T	25	This study
pGF-V2073	pGF-V1895; G12A	25	This study
pGF-V2074	pGF-V1895; G12C	25	This study
pGF-V1958	pGF-V1895; G13A	25	This study
pGF-V2069	pGF-V1895; G13C	25	This study
pGF-V2070	pGF-V1895; G13T	25	This study
pGF-V2059	pGF-V1895; C14T	25	This study
pGF-V2075	pGF-V1895; C14A	25	This study
pGF-V2076	pGF-V1895; C14G	25	This study
pGF-V1959	pGF-V1895; T15G	25	This study
pGF-V2077	pGF-V1895; T15A	25	This study
pGF-V2078	pGF-V1895; T15C	25	This study
pGF-V2060	pGF-V1895; A16G	25	This study
pGF-V2079	pGF-V1895; A16C	25	This study
pGF-V2080	pGF-V1895; A16T	25	This study
pGF-V1960	pGF-V1895; C17A	25	This study
pGF-V2081	pGF-V1895; C17G	25	This study

pGF-V2082	pGF-V1895; C17T	25	This study
pGF-V2061	pGF-V1895; G18A	25	This study
pGF-V1961	pGF-V1895; T19G	25	This study
pGF-V2062	pGF-V1895; A20C	25	This study
pGF-V1962	pGF-V1895; G21A	25	This study
pGF-V2063	pGF-V1895; G22A	25	This study
pGF-V1963	pGF-V1895; G23A	25	This study
pGF-V2064	pGF-V1895; A24C	25	This study
pGF-V1964	pGF-V1895; G25A	25	This study
pGF-V2149[d]	pRS425; *prSNR52::Fn-sgRNA(Kan^R)::SUP4(t)*	25	This study

[a]The (u1') guide DNA sequence was 5'-CGGTGGACTTCGGCTACGTAGGGAG-3' (25 bp example). This was designated as the WT (u1') sequence. The expression cassette for the guide RNA was modeled after a previous study [4]. Briefly, this included 269 bp of the *SNR52* promoter and 20 bp of the *SUP4* terminator. The *Fn*Cas12a guide sequence consisted of a short paired 19 bp repeat (5'-AATTTCTACTGTTGTAGAT-3') [5] that flanked the intended crRNA sequence (typically 25 bp). The entire gRNA expression cassette was synthesized *de novo* (Genscript), cloned into a pUC57 vector (Kan^R), and subcloned to the pRS425 (Amp^R) yeast vector using unique restriction sites.
[b]PCR mutagenesis [6] of the original pGF-V1895 plasmid was used to modify the crRNA length (between 16-27 bp).
[c]PCR mutagenesis was used to introduce substitutions into pGF-V1895. The DNA changes are listed within the table (for the final guide RNA changes, U replaces T). In some instances, direct gene synthesis was used to create the mutational change (e.g., pGF-V2224 and V2225).
[d]The DNA target sequence within Kan^R was 5'-**TTTC**GAGGCCGCGATTAAATTCCAACATG-3'.

Table S3. Oligonucleotides used in this study.

**Oligonucleotide Name**	**DNA Sequence (5' to 3')**
**F1:** Int pr*GAL1/10* +192 F	GGGGTAATTAATCAGCGAAGCGATGATTTTTG
**F2:** Int *Fn*Cas12a Seq #6 F	CAGAAACTCAGATAAGAACCATAACTGGGATAC
**F3:** pr*HIS3* +196 F	GGCCTCCTCTAGTACACTCTATATTTTTTTATGC
**F4:** pr*CCW12* Int F	CGTACAAGTATTTCTCAGGAGTAAAAAAACCGTTTG
**F5:** Int *SpHIS5* F1	GGGAGAACAAGTAATCCAAGTAGACACGGG
**F6:** pr*LYS2* +258 F	CAATAGTTTTGCCAGCGGAATTCCACTTGC
**F7:** pr*LYS2* +636 F	GGTAAGTATGCTCATCAATCGTTCGGACTC
**R1:** Int *Fn*Cas12a Seq #7 R	CACAAGATCAAATCTGATTCTTGACCTTTCTTAGC
**R2:** Int ADH R	CCTGACCTACAGGAAAGAGTTACTCAAGAATAAG
**R3:** Int *SpHIS5* R1	CTGCTTGAATGCAATACCAAGTGCAATAGCAG
**R4:** *HIS3*(t) -151 R	CGCCTCGTTCAGAATGACACGTATAGAATG
**R5:** *SHS1*(t) -192 R	GCCATATTTAAATTTATCCCTACAATTATTTGACACTGTTTG
**R6:** Int *LYS2* +629 R	GTTATGCAATTGGATGGATCGCTTAGCGC
**R7:** *LYS2*(t) -755 R	CGGGCTAAGTATCGATTTGTCTCAACCTGC

Table S4. Quantification of *Fn*Cas12a gene drive strains on agar plates (triplicate)

**GD Strain**	**Plasmid; Guide RNA Sub.**	**Quantification**[a]	**Repeated Activation**[b]
GFY-4625	pGF-V2214; G3U	44 colonies	No
GFY-4625	pGF-V2223; A7G	65 colonies	No
GFY-4626	pGF-V2221; G6C	>99 colonies[c]	Yes (single score)
GFY-4626	pGF-V2056; C8A	>99 colonies	Yes (single score)
GFY-4626	pGF-V2065; U9A	>99 colonies	Yes (single score)
GFY-4626	pGF-V2066; U9G	>99 colonies	Yes (single score)
GFY-4626	pGF-V2071; U10A	77 colonies	Yes (combined score)
GFY-4626	pGF-V1957; C11A	>99 colonies	Yes (single score)
GFY-4626	pGF-V2067; C11U	96 colonies	Yes (combined score)
GFY-4626	pGF-V2058; G12U	>99 colonies	Yes (single score)
GFY-4626	pGF-V2073; G12A	>99 colonies	Yes (single score)
GFY-4626	pGF-V2074; G12C	>99 colonies	Yes (single score)
GFY-4626	pGF-V2069; G13C	>99 colonies	Yes (single score)
GFY-4626	pGF-V2078; U15C	>99 colonies	Yes (single score)
GFY-4626	pGF-V2060; A16G	37 colonies	Yes (combined score)
GFY-4626	pGF-V2080; A16U	>99 colonies	Yes (single score)
GFY-4626	pGF-V1960; C17A	73 colonies	Yes (combined score)
GFY-4626	pGF-V2063; G22A	>99 colonies	Yes (single score)
GFY-4626	pGF-V2064; A24C	>99 colonies	Yes (single score)
GFY-4626	pGF-V1964; G25A	>99 colonies	Yes (single score)

[a]For all other experimental trials, 100 or more colonies were quantified in a single blind fashion. For the six conditions listed, between 37 to 96 colonies were counted and included within the analysis.
[b]For a set of experimental trials (with strain GFY-4626), diploids (maintained from the trial) were subjected to a second independent round of activation as before in order to obtain a larger final colony sample size. For four conditions, the colonies across both trials (GFY-4626) were combined and the total number quantified was presented.
[c]For most conditions, a sample of the total petri dish area was randomly chosen for quantification (sector or bisected segment) from the control (SD-URA-LEU) condition. Typically, between 100 to 300 colonies were quantified.

Figure S1. DNA sequences used in this study.

FnCas12a Gene Drive: (yeast strains GFY-4625, GFY-4626, and GFY-4627)

prHIS3-[u2′]-prGAL-FnCpf1-NLS-ADH1(t)-prMX-CaURA3-MX(t)-[u2′]-HIS3(t)

…GGAAAAACTTATCGAAAGATGACGACTTTTTCTTAATTCTCGTTTTAAGAGCTTGGTGAGCGCTAGGAGTCACTGC
CAGGTATCGTTTGAACACGGCATTAGTCAGGGAAGTCATAACACAGTCCTTTCCCGCAATTTTCTTTTTCTATTACT
CTTGGCCTCCTCTAGTACACTCTATATTTTTTTATGCCTCGGTAATGATTTTCATTTTTTTTTTTCCACCTAGCGGA
TGACTCTTTTTTTTTCTTAGCGATTGGCATTATCACATAATGAATTATACATTATATAAAGTAATGTGATTTCTTCG
AAGAATATACTAAAAAATGAGCAGGCAAGATAAACGAAGGCAAAGTTTTCGCTGTTCGTGTGCGCGTCCTGGGAGTG
ACAGGTTATCAGCAACAACACAGTCATATCCATTCTCAATTAGCTCTACCACAGTGTGTGAACCAATGTATCCAGCA
CCACCTGTAACCAAAACAaTTTTAGAAGTACTTTCACTTTGTAACTGAGCTGTCATTTATATTGAATTTTCAAAAAT
TCTTACTTTTTTTTTGGATGGACGCAAAGAAGTTTAATAATCATATTACATGGCATTACCACCATATACATATCCAT
ATACATATCCATATCTAATCTTACTTATATGTTGTGGAAATGTAAAGAGCCCCATTATCTTAGCCTAAAAAAACCTT
CTCTTTGGAACTTTCAGTAATACGCTTAACTGCTCATTGCTATATTGAAGTACGGATTAGAAGCCGCCGAGCGGGTG
ACAGCCCTCCGAAGGAAGACTCTCCTCCGTGCGTCCTCGTCTTCACCGGTCGCGTTCCTGAAACGCAGATGTGCCTC
GCGCCGCACTGCTCCGAACAATAAAGATTCTACAATACTAGCTTTTATGGTTATGAAGAGGAAAAATTGGCAGTAAC
CTGGCCCCACAAACCTTCAAATGAACGAATCAAATTAACAACCATAGGATGATAATGCGATTAGTTTTTTAGCCTTA
TTTCTGGGGTAATTAATCAGCGAAGCGATGATTTTTGATCTATTAACAGATATATAAATGCAAAAACTGCATAACCA
CTTTAACTAATACTTTCAACATTTTCGGTTTGTATTACTTCTTATTCAAATGTAATAAAAGTATCAACAAAAAATTG
TTAATATACCTCTATACTTTAACGTCAAGGAGAAAAAACTATA**ATGTCTATCTATCAAGAATTCGTTAATAAGTACT**
CTTTGTCAAAGACTTTGAGATTCGAATTGATCCCACAAGGTAAAACATTGGAAAACATCAAGGCTAGAGGTTTGATT
TTGGATGATGAAAAGAGAGCTAAAGATTACAAGAAAGCAAAGCAAATCATCGATAAGTACCATCAATTTTTCATTGA
AGAAATTTTATCTTCAGTTTGTATTTCAGAAGATTTGTTACAAAATTATTCTGATGTTTACTTTAAATTGAAGAAAT
CTGATGATGATAATTTGCAAAAAGATTTTAAATCTGCTAAAGATACTATTAAGAAACAAATTTCAGAATACATTAAA
GATTCTGAAAAGTTTAAAAATTTGTTTAATCAAAATTTGATTGATGCTAAGAAAGGTCAAGAATCAGATTTGATCTT
GTGGTTGAAGCAATCTAAGGATAACGGTATTGAATTGTTTAAAGCTAATTCAGATATTACTGATATCGATGAAGCAT
TAGAAATTATTAAATCTTTTAAAGGTTGGACTACATACTTCAAGGGTTTCCATGAAAACAGAAAGAATGTTTACTCT
TCAAACGATATCCCAACATCAATCATCTATAGAATCGTTGATGATAATTTGCCAAAATTTTTGGAAAATAAGGCTAA
GTACGAATCTTTGAAGGATAAGGCTCCAGAAGCAATTAATTACGAACAAATTAAGAAAGATTTGGCAGAAGAATTGA
CTTTCGATATCGATTACAAGACATCAGAAGTTAACCAAAGAGTTTTCTCTTTGGATGAAGTTTTCGAAATCGCTAAC
TTCAACAACTATTTGAATCAATCAGGTATTACTAAGTTTAATACAATCATCGGTGGTAAATTCGTTAACGGTGAAAA
TACTAAGAGAAAGGGTATTAATGAATACATCAATTTGTACTCTCAACAAATTAATGATAAAACTTTGAAGAAATATA
AAATGTCAGTTTTGTTTAAACAAATTTTATCTGATACAGAATCTAAATCATTTGTTATTGATAAATTGGAAGATGAT
TCAGATGTTGTTACTACAATGCAATCTTTTTACGAACAAATCGCTGCTTTTAAAACTGTTGAAGAAAAATCAATTAA
AGAAACATTGTCTTTGTTGTTCGATGATTTGAAGGCTCAAAAATTGGATTTGTCAAAGATCTATTTCAAGAACGATA
AATCATTGACAGATTTGTCTCAACAAGTTTTCGATGATTACTCTGTTATTGGTACTGCTGTTTTAGAATACATCACA
CAACAAATCGCACCTAAAAATTTGGATAACCCATCTAAGAAAGAACAAGAATTGATCGCTAAGAAAACTGAAAAAGC
AAAGTACTTGTCTTTAGAAACAATTAAATTGGCTTTAGAAGAATTCAATAAGCATAGAGATATCGATAAGCAATGTA
GATTCGAAGAAATTTTGGCAAACTTCGCTGCAATCCCAATGATCTTCGATGAAATCGCTCAAAATAAGGATAATTTG
GCACAAATCTCAATTAAATACCAAAACCAAGGTAAAAAGGATTTGTTACAAGCATCTGCAGAAGATGATGTTAAAGC
TATTAAAGATTTGTTAGATCAAACTAACAATTTGTTGCATAAATTGAAGATCTTTCATATTTCTCAATCAGAAGATA
AAGCTAATATTTTGGATAAAGATGAACATTTTTACTTAGTTTTTGAAGAATGTTATTTTGAATTGGCAAATATTGTT
CCATTGTACAATAAGATCAGAAACTACATCACACAAAAACCATATTCAGATGAAAAGTTTAAATTGAATTTTGAAAA
TTCTACTTTGGCTAACGGTTGGGATAAGAATAAGGAACCAGATAACACAGCAATCTTGTTTATTAAGGATGATAAGT
ACTACTTAGGTGTTATGAATAAGAAAAATAATAAGATTTTTGATGATAAAGCTATTAAAGAAAATAAGGGTGAAGGT
TACAAGAAAATTGTTTACAAATTGTTACCAGGTGCTAATAAGATGTTGCCAAAGGTTTTCTTTTCTGCAAAGTCTAT
TAAATTCTACAACCCATCTGAAGATATTTTAAGAATTAGAAATCATTCAACTCATACTAAAAATGGTTCTCCACAAA
AGGGTTACGAAAAGTTCGAATTCAATATTGAAGATTGTAGAAAGTTTATTGATTTCTACAAGCAATCTATCTCAAAG
CATCCAGAATGGAAGGATTTCGGTTTTAGATTTTCAGATACTCAAAGATACAACTCTATCGATGAATTCTACAGAGA
AGTTGAAAATCAAGGTTACAAATTGACATTTGAAAATATTTCTGAATCATATATTGATTCTGTTGTTAATCAGGGTA
AATTGTATTTGTTCCAAATCTATAATAAGGATTTTTCAGCTTACTCTAAGGGTAGACCAAATTTGCATACTTTGTAC
TGGAAAGCATTGTTCGATGAAAGAAATTTGCAAGATGTTGTTTACAAATTGAACGGTGAAGCTGAATTGTTTTATAG
AAAGCAATCAATCCCAAAGAAAATTACACATCCAGCAAAGGAAGCTATCGCAAATAAGAATAAGGATAACCCAAAGA
AAGAATCCGTTTTCGAATACGATTTGATTAAAGATAAGAGATTCACTGAAGATAAATTTTTCTTTCATTGTCCAATC

ACAATTAATTTCAAGTCTTCAGGTGCTAATAAGTTTAATGATGAAATTAATTTGTTATTGAAAGAAAAAGCAAATGA
TGTTCATATCTTGTCTATCGATAGAGGTGAAAGACATTTGGCTTATTACACTTTAGTTGATGGTAAAGGCAACATTA
TTAAGCAAGATACTTTTAATATTATTGGTAATGATAGAATGAAGACAAACTACCATGATAAATTGGCTGCAATCGAA
AAGGATAGAGATTCAGCAAGAAAGGATTGGAAGAAAATTAATAACATCAAGGAAATGAAGGAAGGTTACTTGTCTCA
AGTTGTTCATGAAATCGCTAAATTAGTTATTGAATACAATGCAATTGTTGTTTTTGAAGATTTGAATTTTGGTTTTA
AAAGAGGTAGATTCAAAGTTGAAAAGCAAGTTTACCAAAAATTGGAAAAGATGTTGATCGAAAAATTGAACTATTTG
GTTTTTAAAGATAACGAATTCGATAAGACTGGTGGTGTTTTGAGAGCTTACCAATTGACTGCACCATTCGAAACTTT
TAAGAAAATGGGTAAACAAACAGGTATCATCTATTACGTTCCAGCTGGTTTTACTTCTAAGATCTGTCCAGTTACAG
GTTTCGTTAACCAATTGTACCCAAAGTACGAATCTGTTTCAAAGTCTCAAGAATTTTTCTCTAAGTTCGATAAGATC
TGTTACAATTTGGATAAAGGTTATTTTGAATTTTCTTTTGATTACAAAAATTTTGGTGACAAAGCTGCAAAGGGTAA
ATGGACTATTGCTTCATTTGGTTCAAGATTGATTAATTTCAGAAACTCAGATAAGAACCATAACTGGGATACTAGAG
AAGTTTACCCAACAAAGGAATTAGAAAAATTGTTGAAGGATTACTCTATCGAATACGGTCATGGTGAATGTATTAAA
GCTGCAATTTGTGGTGAATCAGATAAGAAATTTTTCGCTAAATTGACTTCTGTTTTGAACACAATCTTGCAAATGAG
AAACTCAAAGACTGGTACAGAATTGGATTATTTGATTTCTCCAGTTGCTGATGTTAACGGTAATTTCTTTGATTCAA
GACAAGCACCTAAAAATATGCCACAAGATGCTGATGCAAATGGTGCTTACCATATCGGTTTGAAGGGTTTGATGTTA
TTGGGTAGAATTAAAAATAATCAAGAAGGTAAAAAGTTGAATTTGGTTATTAAAAATGAAGAATACTTCGAATTTGT
TCAAAACAGAAACAATTCAAGAGCTGATCCTAAAAAGAAAAGAAAAGTTTAAggcgcgccacttctaaataagcgaa
tttcttatgatttatgatttttattattaaataagttataaaaaaaataagtgtatacaaattttaaagtgactctt
aggttttaaaacgaaaattcttattcttgagtaactctttcctgtaggtcaggttgctttctcaggtatagtatgag
gtcgctcttattgaccacacctctaccggcagatccgctagggataacagggtaatatAGATCTGTTTAGCTTGCCT
CGTCCCCGCCGGGTCACCCGGCCAGCGACATGGAGGCCCAGAATACCCTCCTTGACAGTCTTGACGTGCGCAGCTCA
GGGGCATGATGTGACTGTCGCCCGTACATTTAGCCCATACATCCCCATGTATAATCATTTGCATCCATACATTTTGA
TGGCCGCACGGCGCGAAGCAAAAATTACGGCTCCTCGCTGCAGACCTGCGAGCAGGGAAACGCTCCCCTCACAGACG
CGTTGAATTGTCCCCACGCCGCGCCCCTGTAGAGAAATATAAAAGGTTAGGATTTGCCACTGAGGTTCTTCTTTCAT
ATACTTCCTTTTAAAATCTTGCTAGGATACAGTTCTCACATCACATCCGAACATAAACAACCATGACAGTCAACACT
AAGACCTATAGTGAGAGAGCAGAAACTCATGCCTCACCAGTAGCACAACGATTATTTCGATTAATGGAACTGAAGAA
AACCAATTTATGTGCATCAATTGATGTTGATACCACTAAGGAATTCCTTGAATTAATTGATAAATTGGGTCCTTATG
TATGCTTAATCAAGACTCATATTGATATAATCAATGATTTTTCCTATGAATCCACTATTGAACCATTATTAGAACTT
TCACGTAAACATCAATTTATGATTTTTGAAGATAGAAAATTTGCTGATATTGGTAATACCGTGAAGAAACAATATAT
TGGTGGAGTTTATAAAATTAGTAGTTGGGCAGATATTACTAATGCTCATGGTGTCACTGGGAATGGAGTAGTTGAAG
GATTAAAACAGGGAGCTAAAGAAACCACCACCAACCAAGAGCCAAGAGGGTTATTGATGTTAGCTGAATTATCATCA
GTGGGATCATTAGCATATGGAGAATATTCTCAAAAAACTGTTGAAATTGCTAAATCCGATAAGGAATTTGTTATTGG
ATTTATTGCCCAACGTGATATGGGTGGACAAGAAGAAGGATTTGATTGGCTTATTATGACACCTGGAGTTGGATTAG
ATGATAAAGGTGATGGATTAGGACAACAATATAGAACTGTTGATGAAGTTGTTAGCACTGGAACTGATATTATCATT
GTTGGTAGAGGATTGTTTGGTAAAGGAAGAGATCCAGATATTGAAGGTAAAAGGTATAGAGATGCTGGTTGGAATGC
TTATTTGAAAAAGACTGGCCAATTATAATCAGTACTGACAATAAAAAGATTCTTGTTTTCAAGAACTTGTCATTTGT
ATAGTTTTTTTATATTGTAGTTGTTCTATTTTAATCAAATGTTAGCGTGATTTATATTTTTTTTCGCCTCGACATCA
TCTGCCCAGATGCGAAGTTAAGTGCGCAGAAAGTAATATCATGCGTCAATCGTATGTGAATGCTGGTCGCTATACTG
CTGTCGATTCGATACTAACGCCGCCATCCAGTTTTCGCTGTTCGTGTGCGCGTCCTGGGAGTGACACCGATTATTTA
AAGCTGCAGCATACGATATATATACATGTGTATATATGTATACCTATGAATGTCAGTAAGTATGTATACGAACAGTA
TGATACTGAAGATGACAAGGTAATGCATCATTCTATACGTGTCATTCTGAACGAGGCGCGCTTTCCTTTTTTCTTTT
TGCTTTTTCTTTTTTTTTCTCTTGAACTCGAGAAAAAAAATATAAAAGAGATGGAGGAACGGAAAAAGTTAGTTGT
GGTGATAGGTGGCAAGTGGTATTCCGTAAGAACAACAAGAAAAGCATTTCATATTATGGCTGAACTGAGCGAA…

Target 1: (yeast strain GFY-4424)

prHIS3-[u1′]-prCCW12-SpHIS5-CDC11(t)-[u1′]-HIS3(t)

…CTCTTGGCCTCCTCTAGTACACTCTATATTTTTTTATGCCTCGGTAATGATTTTCATTTTTTTTTTTCCACCTAGC
GGATGACTCTTTTTTTTTCTTAGCGATTGGCATTATCACATAATGAATTATACATTATATAAAGTAATGTGATTTCT
TCGAAGAATATACTAAAAAATGAGCAGGCAAGATAAACGAAGGCAAAGTTTTCCGGTGGACTTCGGCTACGTAGGGA
GTCAAAGCAAAATAAAAGAAACTTAATACGTTATGCCGTAATGAAGGGCTACCAAAAACGATAATCTCAACTGTAAA
CAGGTACAATGCGGACCCTTTTGCCACAAAACATACATCATTCATTGCCGGAAAAAGAAAGAAGTGAAGACAGCAGT
GCAGCCAGCCATGTTGCGCCAATCTAATTATAGATGCTGGTGCCCTGAGGATGTATCTGGAGCCAGCCATGGCATCA
TGCGCTACCGCCGGATGTAAAATCCGACACGCAAAAGAAAACCTTCGAGGTTGCGCACTTCGCCCACCCATGAACCA
CACGGTTAGTCCAAAAGGGGCAGTTCAGATTCCAGATGCGGGAATTAGCTTGCTGCCACCCTCACCTCACTAACGCT
GCGGTGTGCGGATACTTCATGCTATTTATAGACGCGCGTGTCGGAATCAGCACGCGCAAGAACCAAATGGGAAAATC

GGAATGGGTCCAGAACTGCTTTGAGTGCTGGCTATTGGCGTCTGATTTCCGTTTTGGGAATCCTTTGCCGCGCGCCC
CTCTCAAAACTCCGCACAAGTCCCAGAAAGCGGGAAAGAAATAAAACGCCACCAAAAAAAAAAAAAATAAAAGCCAAT
CCTCGAAGCGTGGGTGGTAGGCCCTGGATTATCCCGTACAAGTATTTCTCAGGAGTAAAAAAACCGTTTGTTTTGGA
ATTCCCCATTTCGCGGCCACCTACGCCGCTATCTTTGCAACAACTATCTGCGATAACTCAGCAAATTTTGCATATTC
GTGTTGCAGTATTGCGATAATGGGAGTCTTACTTCCAACATAACGGCAGAAAGAAATGTGAGAAAATTTTGCATCCT
TTGCCTCCGTTCAAGTATATAAAGTCGGCATGCTTGATAATCTTTCTTTCCATCCTACATTGTTCTAATTATTCTTA
TTCTCCTTTATTCTTTCCTAACATACCAAGAAATTAATCTTCTGTCATTCGCTTAAACACTATATCAATAATGAGGA
GGGCTTTTGTAGAAAGAAATACGAACGAAACGAAAATCAGCGTTGCCATCGCTTTGGACAAAGCTCCCTTACCTGAA
GAGTCGAATTTTATTGATGAACTTATAACTTCCAAGCATACAAACCAAAAGGGAGAACAAGTAATCCAAGTAGACAC
GGGAATTGGATTCTTGGATCACATGTATCATGCACTGGCTAAACATGCAGGCTGGAGCTTACGACTTTACTCAAGAG
GTGATTTAATCATCGATGATCATCACACTGCAGAAGATACTGCTATTGCACTTGGTATTGCATTCAAGCAGGCTATG
AGTAACTTTGCCGGCGTTAAAAGATTTGGACATGCTTATTGTCCACTTGACGAAGCTCTTTCTAGAAGCGTAGTTGA
CTTGTCGGGACGGCCCTATGCTGTTATCGATTTGGGATTAAAGCGTGAAAAGGTTGGGGAATTGTCCTGTGAAATGA
TCCCTCACTTACTATATTCCTTTTCGGTAGCAGCTGGAATTACTTTGCATGTTACCTGCTTATATGGTAGTAATGAC
CATCATCGTGCTGAAAGCGCTTTTAAATCTCTGGCTGTTGCCATGCGCGCGGCTACTAGTCTTACTGGAAGTTCTGA
AGTCCCAAGCACGAAGGGAGTGTTGTAAGTCCGCTTTTGGCTTCCTCACTTATTTCTTCTTTCTCTATATATATAAA
GAGTGAGTGTTGTATATAAGTAAATACATCTGGTATATTATTTTTTTTTTTTTCTTCATTCTTAAAAAGTATTAATAT
CGATCAGCAAAAAAAAATTAACAAAAAAGTTCCTTATTATATCTGCGTAGAAGTACTTATTTCTGCTCCACCTTTGG
AGTATTTTTCCAAAATTGTGATGCCAAATGAGTAATGAAATAGAATTTCTTGTGTGGATCGTCATTATCGATTTGGT
TTGGACAGTTAAAGGAGAATTTTGGAAAGACCAGAATTTTCCGGTGGACTTCGGCTACGTAGGGAGTGACACCGATT
ATTTAAAGCTGCAGCATACGATATATATACATGTGTATATATGTATACCTATGAATGTCAGTAAGTATGTATACGAA
CAGTATGATACTGAAGATGACAAGGTAATGCATCATTCTATACGTGTCATTCTGAACGAGGCGCGC…

Target 2: (yeast strain GFY-4425)

prHIS3-[u1′]-prCDC12-mCherry-SHS1(t)-prCCW12-SpHIS5-CDC11(t)-[u1′]-HIS3(t)

…CTCTTGGCCTCCTCTAGTACACTCTATATTTTTTTATGCCTCGGTAATGATTTTCATTTTTTTTTTTCCACCTAGC
GGATGACTCTTTTTTTTTCTTAGCGATTGGCATTATCACATAATGAATTATACATTATATAAAGTAATGTGATTTCT
TCGAAGAATATACTAAAAAATGAGCAGGCAAGATAAACGAAGGCAAAGTTTTCCGGTGGACTTCGGCTACGTAGGGA
GTGGGGCAGCGCCCTGTTTTTCATTAATGTAGTCAGCAATGTCAAGATTCAACGCCAAGTCTGGTTCAGCAAGTGAC
ATTCTGCAAGCTCTTTGAATCTTCCTCAAAAGAGGATTGCCCAAGGCTTGAGGTTTCCTGACGGGCAACTCAGACAA
ATATATGCTATGTGAGTGCGGATGGGACATGATGCAGTATCACGATTAGCAATTCAGCTATGAGTTATGTTGCTCTT
TGTTTTGTTTATGGAAATTGTCCTATGGTAAGTCTCTTTTTTTTGCAATCGTGATTACAGAAAAAAAACAGGGCGCT
GGAAAAGTGAAGAATCCGAAATTTTTTTCGAAATCACCATTGTTTGTTTTGAGTAGATCAAAGTCTTGAAAGGTGCA
GCAAGATATAGGATCTTGACCTGAAGAGTATTGATAACGAACTACATCACATATTGTATCAAATAATGGTGAGCAAG
GGCGAGGAGGATAACATGGCCATCATCAAGGAGTTCATGCGCTTCAAGGTGCACATGGAGGGCTCCGTGAACGGCCA
CGAGTTCGAGATCGAGGGCGAGGGCGAGGGCCGCCCCTACGAGGGCACCCAGACCGCCAAGCTGAAGGTGACCAAGG
GTGGCCCCCTGCCCTTCGCCTGGGACATCCTGTCCCCTCAGTTCATGTACGGCTCCAAGGCCTACGTGAAGCACCCC
GCCGACATCCCCGACTACTTGAAGCTGTCCTTCCCCGAGGGCTTCAAGTGGGAGCGCGTGATGAACTTCGAGGACGG
CGGCGTGGTGACCGTGACCCAGGACTCCTCCCTGCAGGACGGCGAGTTCATCTACAAGGTGAAGCTGCGCGGCACCA
ACTTCCCCTCCGACGGCCCCGTAATGCAGAAGAAGACCATGGGCTGGGAGGCCTCCTCCGAGCGGATGTACCCCGAG
GACGGCGCCCTGAAGGGCGAGATCAAGCAGAGGCTGAAGCTGAAGGACGGCGGCCACTACGACGCTGAGGTCAAGAC
CACCTACAAGGCCAAGAAGCCCGTGCAGCTGCCCGGCGCCTACAACGTCAACATCAAGTTGGACATCACCTCCCACA
ACGAGGACTACACCATCGTGGAACAGTACGAACGCGCCGAGGGCCGCCACTCCACCGGCGGCATGGACGAGCTGTAC
AAGTAAGTTGTATCTGTACAAAATCCAAAGCTGAGCAAATAAATAAATAAATAAATGTATAAGTTACCGAACGGGGG
TATTTTTACTTTTGATCAAAAATTTATGTACCAACTACAAAGTTTCCTCAGCACAGCCTTCAAGAAGGGAACACACA
TACAAACAGTGTCAAATAATTGTAGGGATAAATTTAAATATGGCATAAACTAAATAAGTAGAGCATGAAAAAACTGC
AAAATCCAAAAAGTAAAAACGAAGGTCAGAAAGTAAAGCAAAAGAAAATTAATAAAGCAATACTAAATCTATCATGA
TTTCCCGTAACTTCCATTAAAGCTGTAACCAGATTTACTCCTACTGTTTGAGCCTCTAACGCCTAATGGATTTTTAG
AGAAGCTCAACCTGATACCTCCTTTGTTGTTGAGGGAAGGGCGGGGGTGAGGTAGTTGACTACCATATAATTCTGCC
AATGCTCTAGTGGCAAAGCTAACATCCTCACAAAGCAAAATAAAAGAAACTTAATACGTTATGCCGTAATGAAGGGC
TACCAAAAACGATAATCTCAACTGTAAACAGGTACAATGCGGACCCTTTTGCCACAAAACATACATCATTCATTGCC
GGAAAAAGAAAGAAGTGAAGACAGCAGTGCAGCCAGCCATGTTGCGCCAATCTAATTATAGATGCTGGTGCCCTGAG
GATGTATCTGGAGCCAGCCATGGCATCATGCGCTACCGCCGGATGTAAAATCCGACACGCAAAAGAAAACCTTCGAG
GTTGCGCACTTCGCCCACCCATGAACCACACGGTTAGTCCAAAAGGGGCAGTTCAGATTCCAGATGCGGGAATTAGC
TTGCTGCCACCCTCACCTCACTAACGCTGCGGTGTGCGGATACTTCATGCTATTTATAGACGCGCGTGTCGGAATCA

GCACGCGCAAGAACCAAATGGGAAAATCGGAATGGGTCCAGAACTGCTTTGAGTGCTGGCTATTGGCGTCTGATTTC
CGTTTTGGGAATCCTTTGCCGCGCGCCCCTCTCAAAACTCCGCACAAGTCCCAGAAAGCGGGAAAGAAATAAAACGC
CACCAAAAAAAAAAAAATAAAAGCCAATCCTCGAAGCGTGGGTGGTAGGCCCTGGATTATCCCGTACAAGTATTTCT
CAGGAGTAAAAAAACCGTTTGTTTTGGAATTCCCCATTTCGCGGCCACCTACGCCGCTATCTTTGCAACAACTATCT
GCGATAACTCAGCAAATTTTGCATATTCGTGTTGCAGTATTGCGATAATGGGAGTCTTACTTCCAACATAACGGCAG
AAAGAAATGTGAGAAAATTTTGCATCCTTTGCCTCCGTTCAAGTATATAAAGTCGGCATGCTTGATAATCTTTCTTT
CCATCCTACATTGTTCTAATTATTCTTATTCTCCTTTATTCTTTCCTAACATACCAAGAAATTAATCTTCTGTCATT
CGCTTAAACACTATATCAATA**ATGAGGAGGGCTTTTGTAGAAAGAAATACGAACGAAACGAAAATCAGCGTTGCCAT**
CGCTTTGGACAAAGCTCCCTTACCTGAAGAGTCGAATTTTATTGATGAACTTATAACTTCCAAGCATACAAACCAAA
AGGGAGAACAAGTAATCCAAGTAGACACGGGAATTGGATTCTTGGATCACATGTATCATGCACTGGCTAAACATGCA
GGCTGGAGCTTACGACTTTACTCAAGAGGTGATTTAATCATCGATGATCATCACACTGCAGAAGATACTGCTATTGC
ACTTGGTATTGCATTCAAGCAGGCTATGAGTAACTTTGCCGGCGTTAAAAGATTTGGACATGCTTATTGTCCACTTG
ACGAAGCTCTTTCTAGAAGCGTAGTTGACTTGTCGGGACGGCCCTATGCTGTTATCGATTTGGGATTAAAGCGTGAA
AAGGTTGGGGAATTGTCCTGTGAAATGATCCCTCACTTACTATATTCCTTTTCGGTAGCAGCTGGAATTACTTTGCA
TGTTACCTGCTTATATGGTAGTAATGACCATCATCGTGCTGAAAGCGCTTTTAAATCTCTGGCTGTTGCCATGCGCG
CGGCTACTAGTCTTACTGGAAGTTCTGAAGTCCCAAGCACGAAGGGAGTGTTGTAAGTCCGCTTTTGGCTTCCTCAC
TTATTTCTTCTTTCTCTATATATATAAAGAGTGAGTGTTGTATATAAGTAAATACATCTGGTATATTATTTTTTTTT
TTTCTTCATTCTTAAAAAGTATTAATATCGATCAGCAAAAAAAAATTAACAAAAAAGTTCCTTATTATATCTGCGTA
GAAGTACTTATTTCTGCTCCACCTTTGGAGTATTTTTCCAAAATTGTGATGCCAAATGAGTAATGAAATAGAATTTC
TTGTGTGGATCGTCATTATCGATTTGGTTTGGACAGTTAAAGGAGAATTTTGGAAAGACCAGAAT**TTTCCGGTGGAC**
TTCGGCTACGTAGGGAGTGACACCGATTATTTAAAGCTGCAGCATACGATATATATACATGTGTATATATGTATACC
TATGAATGTCAGTAAGTATGTATACGAACAGTATGATACTGAAGATGACAAGGTAATGCATCATTCTATACGTGTCA
TTCTGAACGAGGCGCGC…

WT (u1′) guide construct: (plasmid pGF-V1895)

ACTAGTtcactaaagggaacaaaagctggagcttctttgaaaagataatgtatgattatgctttcactcatatttat
acagaaacttgatgttttctttcgagtatatacaaggtgattacatgtacgtttgaagtacaactctagattttgta
gtgccctcttgggctagcggtaaaggtgcgcatttttttcacaccctacaatgttctgttcaaaagattttggtcaaa
cgctgtagaagtgaaagttggtgcgcatgtttcggcgttcgaaacttctccgcagtgaaagataaatgatc**AATTTC**
TACTGTTGTAGATCGGTGGACTTCGGCTACGTAGGGAGAATTTCTACTGTTGTAGATTTTTTTTGTTTTTTATGTCT
tcgagtcatgtaattagttatgtcacgc**GCGGCCGC**

Kan^R guide construct: (plasmid pGF-V2149)

ACTAGTtcactaaagggaacaaaagctggagcttctttgaaaagataatgtatgattatgctttcactcatatttat
acagaaacttgatgttttctttcgagtatatacaaggtgattacatgtacgtttgaagtacaactctagattttgta
gtgccctcttgggctagcggtaaaggtgcgcatttttttcacaccctacaatgttctgttcaaaagattttggtcaaa
cgctgtagaagtgaaagttggtgcgcatgtttcggcgttcgaaacttctccgcagtgaaagataaatgatc**AATTTC**
TACTGTTGTAGATGAGGCCGCGATTAAATTCCAACATGAATTTCTACTGTTGTAGATTTTTTTTGTTTTTTATGTCT
tcgagtcatgtaattagttatgtcacgc**GCGGCCGC**

(u1′) Guide Length 27 (plasmid pGF-V1941)

ACTAGTtcactaaagggaacaaaagctggagcttctttgaaaagataatgtatgattatgctttcactcatatttat
acagaaacttgatgttttctttcgagtatatacaaggtgattacatgtacgtttgaagtacaactctagattttgta
gtgccctcttgggctagcggtaaaggtgcgcatttttttcacaccctacaatgttctgttcaaaagattttggtcaaa
cgctgtagaagtgaaagttggtgcgcatgtttcggcgttcgaaacttctccgcagtgaaagataaatgatc**AATTTC**
TACTGTTGTAGATCGGTGGACTTCGGCTACGTAGGGAGTGAATTTCTACTGTTGTAGATTTTTTTTGTTTTTTATGT
CTtcgagtcatgtaattagttatgtcacgc**GCGGCCGC**

(u1′) Guide Length 26 (plasmid pGF-V1942)

ACTAGTtcactaaagggaacaaaagctggagcttctttgaaaagataatgtatgattatgctttcactcatatttat
acagaaacttgatgttttctttcgagtatatacaaggtgattacatgtacgtttgaagtacaactctagattttgta
gtgccctcttgggctagcggtaaaggtgcgcatttttttcacaccctacaatgttctgttcaaaagattttggtcaaa
cgctgtagaagtgaaagttggtgcgcatgtttcggcgttcgaaacttctccgcagtgaaagataaatgatc**AATTTC**

TACTGTTGTAGATCGGTGGACTTCGGCTACGTAGGGAGTAATTTCTACTGTTGTAGATTTTTTTTGTTTTTTATGTC
Ttcgagtcatgtaattagttatgtcacgc**GCGGCCGC**

Figure S2. Protocol for yeast chromosomal DNA extraction and purification

1. Grow yeast culture overnight in 5 mL of YPD at 30°C.
2. Spin tubes in clinical centrifuge for 3 minutes.
3. Remove media and save pellet.
4. Resuspend into 500 μL of Genomic Solution #1 (1 M sorbitol, 0.1 M Na2EDTA pH 7.5). Vortex briefly to mix.
5. Add 5 μL of Zymolyase 100T solution (25 mg/mL in 50% glycerol, store at 4°C).
6. Incubate at 37°C for 1-2 hours.
7. Spin for 11,000 rpm for 1 minute.
8. Pipette off the solution and keep pellet.
9. Resuspend in 500 μL of Genomic Solution #2 (50 mL Tris-Cl pH 7.4, 20 mM Na2EDTA). Vortex for 30 seconds to mix.
10. Add 50 μL of 10% SDS solution.
11. Vortex 5 seconds to mix.
12. Incubate at 65°C for 35 minutes in heating block.
13. Add 200 μL of 5M KOAc and mix by inversion 10 times.
14. Incubate on ice for 1 hour.
15. Spin at max speed for 5 minutes.
16. Pour into a fresh tube that contains 550 μL of isopropanol.
17. Mix by inversion.
18. Let sit for 30 seconds.
19. Spin for 1 minute at max speed.
20. Pour off isopropanol and keep pellet.
21. Spin a second time for 30 seconds at 8,000 rpm.
22. Pipette off the remaining isopropanol and wait 5 minutes.
23. Add 300 μL of TE solution (10 mM Tris-Cl pH 7.4 and 1 mM Na_2EDTA).
24. Incubate for 15 minutes at room temperature.
25. Use a pipette to resuspend the pellet.
26. Incubate at room temperature another 15 minutes.
27. Fully resuspend the pellet.
28. Add 1.5 μL of RNAaseA solution (stored at -20°C at 10 mg/mL solution in water; boiled for 10 minutes initially, then frozen).
29. Incubate at 37°C for 30 minutes.
30. Add 30 μL of 3 M sodium acetate and vortex.
31. Add 200 μL of isopropanol and invert.
32. Allow pellet to fall to bottom of tube.
33. Spin at max for 1 minute.
34. Remove isopropanol solution.
35. Spin at 8,000 rpm for 30 seconds.
36. Pipette off isopropanol solution. Allow pellet to dry for 5 minutes at room temperature.
37. Add 100 μL of TE solution. Allow incubation for 15 minutes at room temperature.
38. Resuspend pellet into TE.
39. Store at -20°C.

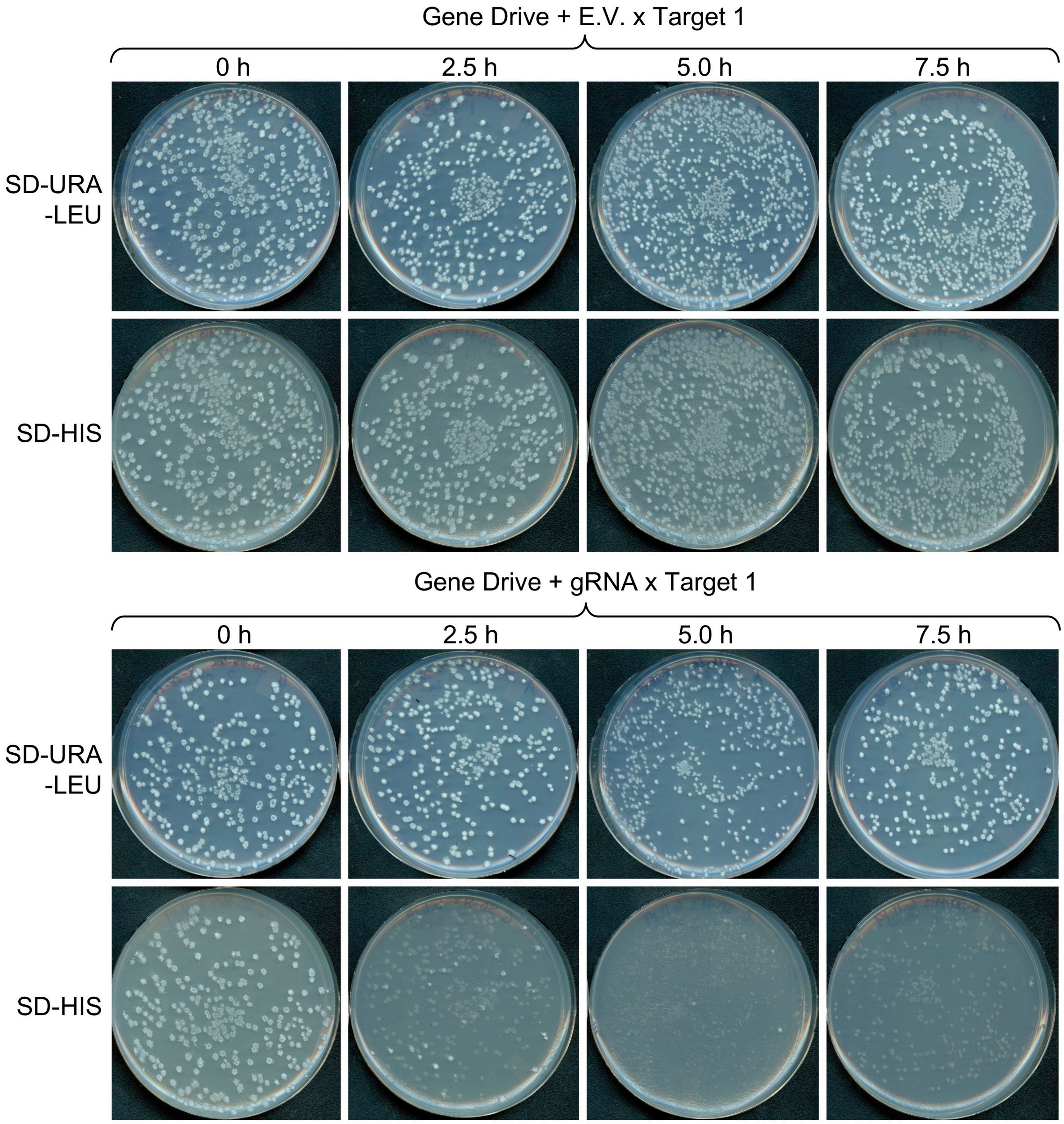

Figure S3. Unmodified agar plate images illustrating gene drive diploid yeast colonies. Scanned images (from Fig. 2) were cropped to illustrate individual plates (separated by white lines); no additional alterations have been performed.

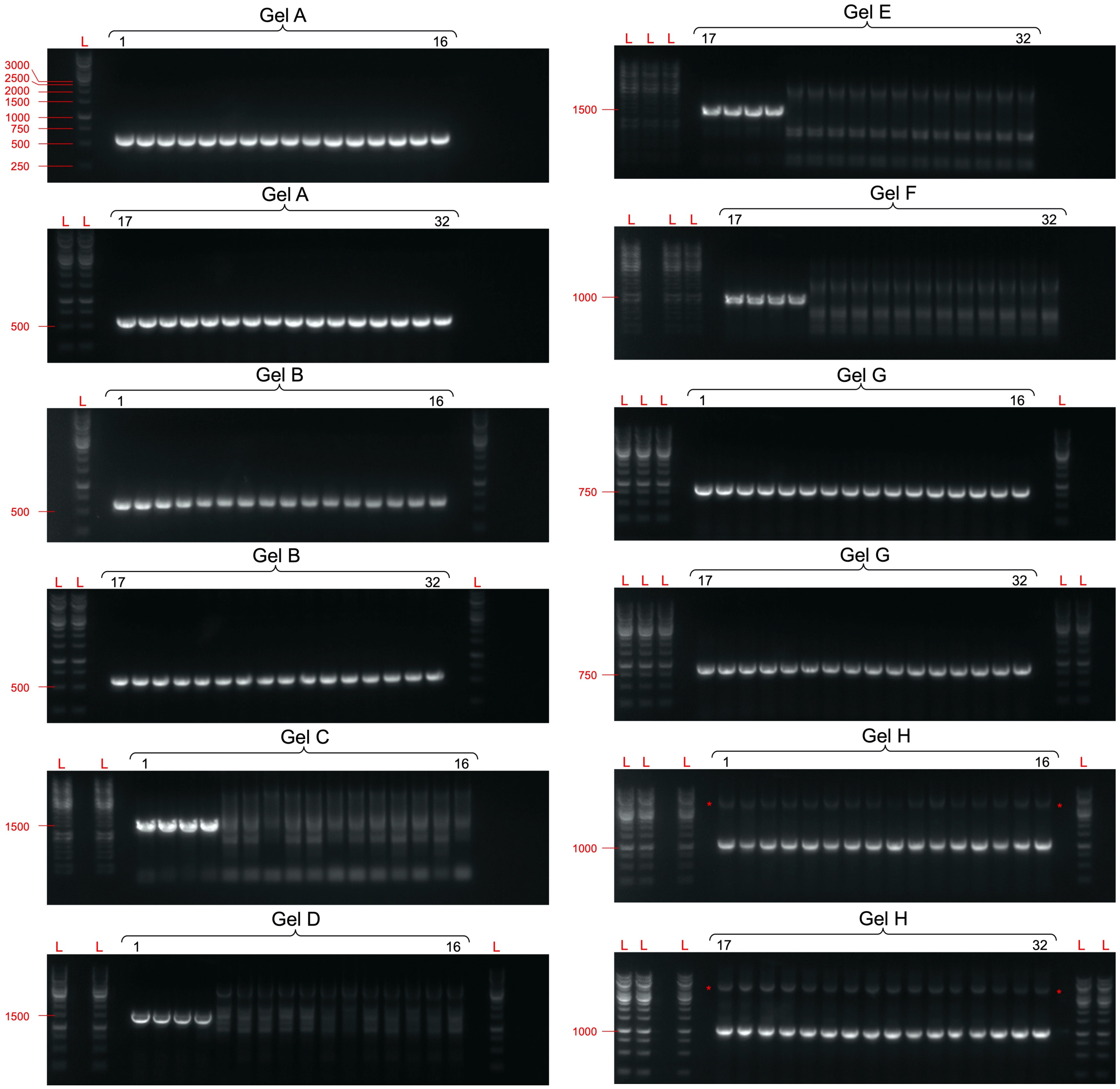

Figure S4. Unmodified DNA gel images illustrating diagnostic PCRs of yeast chromosomal preparations. Individual images (from Fig. 3B) were cropped and labeled for clarify; no additional alterations have been performed. Each DNA gel (A, B, C, etc.) corresponds to the same labels provided within Fig. 3. L, DNA ladders (the same molecular ladders were used for all experiments; labels of the nearest marker are shown that correspond to Fig. 3B). Red asterisks, position of (expected) alternative PCR product from the *LYS2* locus for Gel H (5,573 bp) from diploid genomes. Note, PCR conditions were optimized for the smaller fragment.

REFERENCES

1. **Brachman CB, Davies A, Cost GJ, Caputo E, Li J *et al.*** Designer deletion strains derived from *Saccharomyces cerevisiae* S288C: a useful set of strains and plasmids for PCR-mediated gene disruption and other applications. *Yeast (Chichester, England)* 1998; 14: 115-132 DOI: 10.1002/(SICI)1097-0061(19980130)14:2<115::AID-YEA204>3.0.CO;2-2

2. **Yan Y, Finnigan GC.** Analysis of CRISPR gene drive design in budding yeast. *Access Microbiol* 2019; 1(9): e000059 DOI: 10.1099/acmi.0.000059

3. **Christianson TW, Sikorski RS, Dante M, Shero JH, Hieter P.** Multifunctional yeast high-copy-number shuttle vectors. *Gene* 1992; 110: 119-122 DOI: 10.1016/0378-1119(92)90454-w

4. **DiCarlo JE**, **Norville JE**, **Mali P**, **Rios X**, **Aach J** ***et al.*** Genome engineering in *Saccharomyces cerevisiae* using CRISPR-Cas systems. *Nucleic Acids Res* 2013; 41:4336-4343 DOI: 10.1093/nar/gkt135

5. **Swiat MA, Dashko S, den Ridder M, Wijsman M, van der Oost J *et al.*** *Fn*Cpf1: a novel and efficient genome editing tool for *Saccharomyces cerevisiae*. *Nucleic Acids Res* 2017; 45(21): 12585-12598 DOI: 10.1093/nar/gkx1007

6. **Zheng L, Baumann U, Reymond JL**. An efficient one-step site-directed and site-saturation mutagenesis protocol. *Nucleic Acids Res* 2004; 32(14): e115 DOI: 10.1093/nar/gnh110
